# Essential Oils of Aromatic Plant Species from the Atlantic Rainforest Exhibit Extensive Chemical Diversity and Antimicrobial Activity

**DOI:** 10.3390/antibiotics11121844

**Published:** 2022-12-19

**Authors:** Crislene V. Perigo, Lenita L. Haber, Roselaine Facanali, Maria A. R. Vieira, Roseli B. Torres, Luís C. Bernacci, Elsie F. Guimarães, João B. Baitello, Marcos E. G. Sobral, Vera Quecini, Marcia Ortiz M. Marques

**Affiliations:** 1 Instituto Agronômico, Campinas 13075-630, Brazil; 2 Vegetables Research Center, Brazilian Agricultural Research Corporation, Brasília 70351-970, Brazil; 3 Instituto de Pesquisas Jardim Botânico do Rio de Janeiro, Rio de Janeiro 22460-030, Brazil; 4 Instituto Florestal do Estado de São Paulo, São Paulo 02377-000, Brazil; 5 Natural Sciences Department, Campus Dom Bosco, Universidade Federal de São João del-Rei, São João del Reio 36301-160, Brazil; 6 Grape and Wine Research Center, Brazilian Agricultural Research Corporation, Bento Gonçalves 95701-008, Brazil

**Keywords:** ADME, biological activity, GC-MS, network analyses, terpene, volatiles

## Abstract

Microbial resistance, caused by the overuse or inadequate application of antibiotics, is a worldwide crisis, increasing the risk of treatment failure and healthcare costs. Plant essential oils (EOs) consist of hydrophobic metabolites with antimicrobial activity. The antimicrobial potential of the chemical diversity of plants from the Atlantic Rainforest remains scarcely characterized. In the current work, we determined the metabolite profile of the EOs from aromatic plants from nine locations and accessed their antimicrobial and biocidal activity by agar diffusion assays, minimum inhibitory concentration, time-kill and cell-component leakage assays. The pharmacokinetic properties of the EO compounds were investigated by in silico tools. More than a hundred metabolites were identified, mainly consisting of sesqui and monoterpenes. Individual plants and botanical families exhibited extensive chemical variations in their EO composition. Probabilistic models demonstrated that qualitative and quantitative differences contribute to chemical diversity, depending on the botanical family. The EOs exhibited antimicrobial biocidal activity against pathogenic bacteria, fungi and multiple predicted pharmacological targets. Our results demonstrate the antimicrobial potential of EOs from rainforest plants, indicate novel macromolecular targets, and contribute to highlighting the chemical diversity of native species.

## 1. Introduction

Antimicrobial resistance is the main cause of relapsing infections and treatment failure in microbe-induced pathogenesis, leading to higher rates of patient morbidity and mortality, but also imposing increased costs to healthcare [1]. The selective pressure enforced by the overuse and/or misuse of antimicrobials triggers genetic and metabolic modifications in pathogenic microorganisms that allow them to extrude or detoxify multiple drugs, giving rise to Multidrug-Resistant (MDR) pathogens [1]. The molecular mechanisms underlying drug resistance are classified into three main groups: (i) reduction in the intracellular concentration of the antimicrobial agent; (ii) molecular modifications of the antimicrobial target; and (iii) inactivation of the antimicrobial molecule [2]. The evolution of pathogenic microorganisms shuffles and combines these general mechanisms to overcome the mode of action of several classes of antimicrobial compounds. Thus, new therapeutic molecules are continuously sought after to combat MDR pathogens.

Several plant compounds and mixtures of compounds exhibit antimicrobial potential, including Essential Oils (EOs) [3]. EOs are an important part of the volatile organic metabolites, produced by four major biosynthetic routes: the shikimate/phenylalanine, the mevalonic acid, the methylerythritol phosphate and lipoxygenase pathways [4,5,6,7]. EOs consist of hydrophobic metabolites stored in and released by specialized secretory structures of the plants, involved in a wide range of biotic interactions in the natural environment, including with herbivores and their parasitoids, pollinators, and other plants [4,6,7]. Chemically, EOs consist of complex blends of terpenoids, benzenoids/phenylpropanoids, volatile carotenoid derivatives, and methylated volatiles [6,7]. The hydrophobicity and variable degree of reactivity of EO metabolites make them interesting therapeutic products to be used against pathogenic microorganisms, alone or in combination with traditional antibiotics [8,9,10]. However, a large portion of the plants’ chemical diversity remains unexplored, as most studies have focused on domesticated species. The flora in the Brazilian portion of the Atlantic Rainforest is considered one of the richest in the world, consisting of more than forty-thousand species, with nearly half of them being endemic [11]. The biome is also one of the primary biodiversity hotspots in the world, with approximately twenty-thousand species [11]. The plants in the Atlantic Rainforest exhibit high inter- and intra-specific genetic variation, which, coupled with the distinct environmental conditions, allows them to produce hundreds of thousands of distinct specialized metabolites [12]. The chemical diversity of the EOs from rainforest plants remains scarcely characterized. Comprehensive chemical characterization of the EOs from undomesticated species may discover novel aspects of the plants metabolic diversity and contribute alternative compounds for green chemistry applications, including the design of novel pharmaceuticals.

The current work aimed at investigating the chemical composition and antimicrobial potential of the EOs from plant species found in the Atlantic Rainforest. The results demonstrate expressive inter- and intra-specific chemical variation, significant antimicrobial activity and interesting pharmacokinetic characteristics and macromolecular pharmacological targets of the EO compounds. These findings can contribute to their use in pharmaceutical applications.

## 2. Results

### 2.1. Botanical and Chemical Characterization

**Figure 1:**
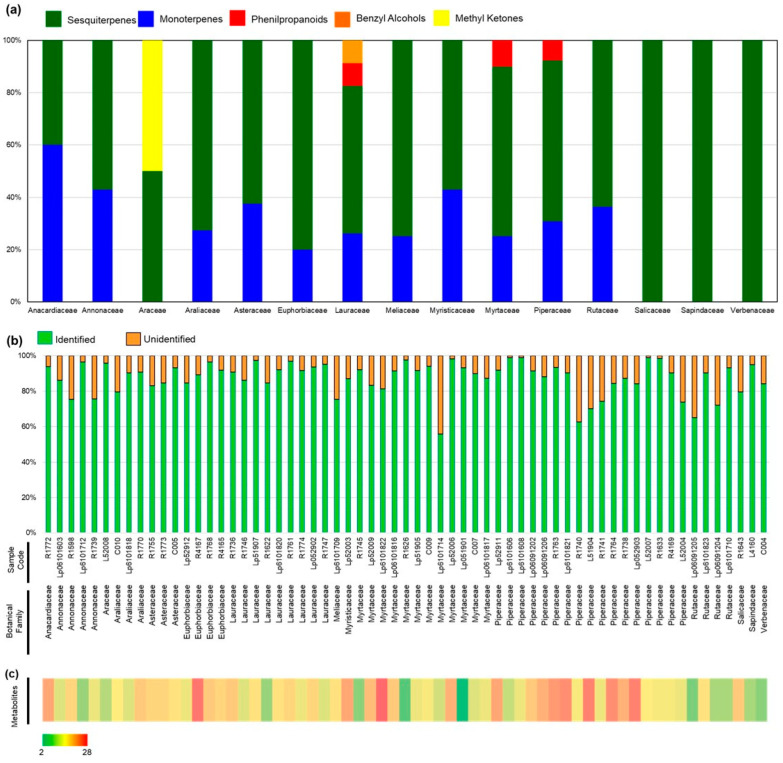
Summary of the metabolite profiles of aromatic plants from the Atlantic Rainforest. (**a**) Percentage of identified metabolites per chemical class for each botanical family. (**b**) Percentage of unidentified features per EO. (**c**) Chemical complexity of EO samples represented as heatmap.

Fifty distinct aromatic plant species, belonging to fifteen botanical families, were identified in nine locations of the Atlantic rainforest in the State of São Paulo (Table S1, Figure S1). The number of families with associated traditional use were found in coastal locations (Ubatuba and Pariquera-Açu) and the transition region in Votuporanga (Figures S1 and S2). The metabolite profile of 63 EO samples was determined by GC-MS (Figure 1, Table 1). The oil yield was highly variable, ranging between 0.004 and 2.88%, with shrubs producing approximately 1.6 times higher contents than trees (Figure S3). The highest EO contents were found in Pipearaceae shrubs (Figure S3). The biological metabolite variation ranged between trace amounts (≤0.05) and 94.46%. The complete chemical data and their associated metadata are deposited at the National Metabolomics Repository, under identifier ST000606.

**Figure 2:**
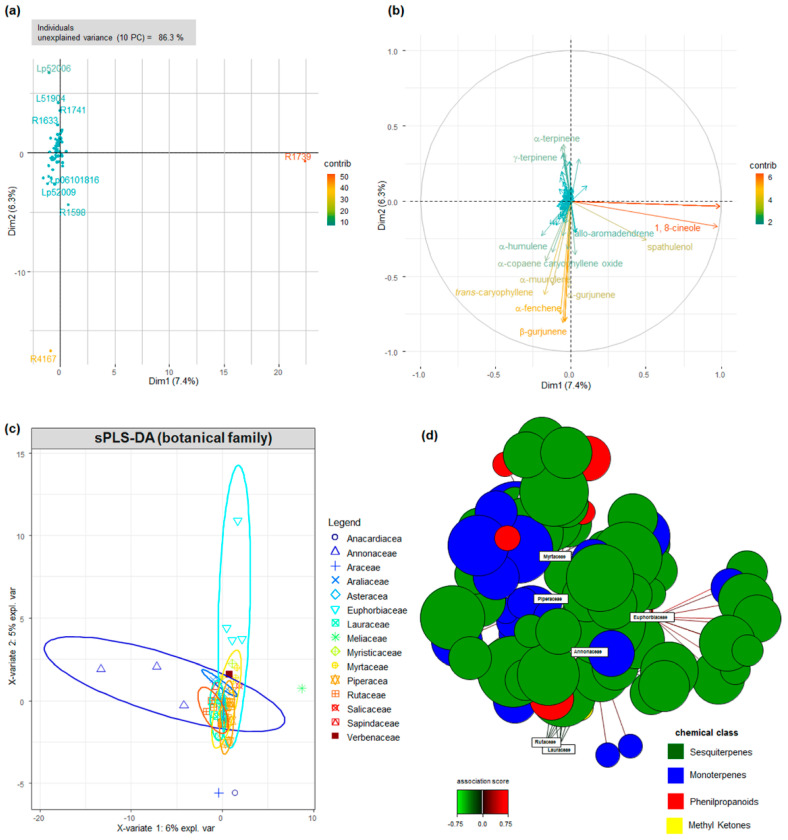
Multivariate analyses of the chemical composition of the Eos. Principal Component Analysis (PCA) of the chemical profile of the Eos, represented as (**a**) individual (EO sample) and (**b**) variable (metabolite contents) contributions to the total variance. Contribution is a variable scaled version of the squared correlation between individual profiles/variables and component axes, represented as color scale. (**c**) Sparse Partial Least Square (sPLS) classification of the EO chemical profiles using the botanical families as discriminant (DA). Confidence ellipses at 95% were generated by 100 times bootstrapping and are color-coded. (**d**) Relevance network for metabolite and botanical family association at 75% threshold. Chemical classes and sPLS−DA association are represented by colors.

A total of 113 metabolites were identified, consisting mostly of monoterpenes (MT) (29%) and sesquiterpenes (ST) (56%), along with phenylpropanoids (PP) (7.5%), benzyl alcohols (BA) (3%) and ketones (MK) (3%) (Figure 1). More than 50% (63/113) of the metabolites were present as major components (Table 1) and most metabolites in the EOs chemical composition (≥80%) were identified in all samples (50 out of 63, 79.4%) (Table 1, Figure 1 and Figure 2). The percentage of unidentified metabolites ranged between 1.12% (Lp6101606, *Piper aduncum*) and 44.2% (Lp6101714, *Eugenia myrcianthes*) of the total (Figure 1). The number of metabolites per sample ranged between 2 (Lp051901, *Myrcia spectabilis*) and 28 (Lp6101822, *Campomanesia guavirota*) (Figure 1).

The identified metabolites were classified in monoterpenes (MT) (29%), sesquiterpenes (ST) (56%), phenylpropanoids (PP) (7.5%), benzenoids (BA) (3%), and ketones (MK) (3%) (Table 1, Figures S4–S6). The most frequent compound was α-pinene, followed by bicyclogermacrene, germacrene D and *trans*-caryophyllene (Table 1). The least frequent metabolites, present above trace levels, were geraniol and methyl-geranate (Table S1). Coastal locations (Pariquera-Açu and Ubatuba) exhibited higher botanical diversity of aromatic species, alongside the plateau sites in Campinas and Votuporanga (Figures S1 and S2).

Multivariate analyses demonstrated that a large portion (86.3%) of the variation in the chemical composition of the EOs remained unexplained, even when considering ten principal components (Figure 2 and Figure S4). A sparse Partial Least Squares Regression (sPLS) approach, using the botanical families as discriminant variables (DA, discriminant analysis), was employed to reduce data dimensionality (Figure 2). The supervised classification was not enough to clearly attribute the EOs’ chemical composition to a given botanical family, as a wide range of metabolites was shared by the investigated individuals. We hypothesized that the unbalanced nature of the data—that is, the uneven frequency of botanical families among the locations—could have contributed to the poor prediction performance of the method. Therefore, simulations with balanced data were carried out (Figure S4), although they were not sufficient in increasing the classification performance of the method, suggesting the existence of high intra-specific chemical variation. The presence of family-specific compounds, such as geraniol in Annonaceae and *n*-octane in Euphorbiaceae, contributed to group separation.

**Figure 3:**
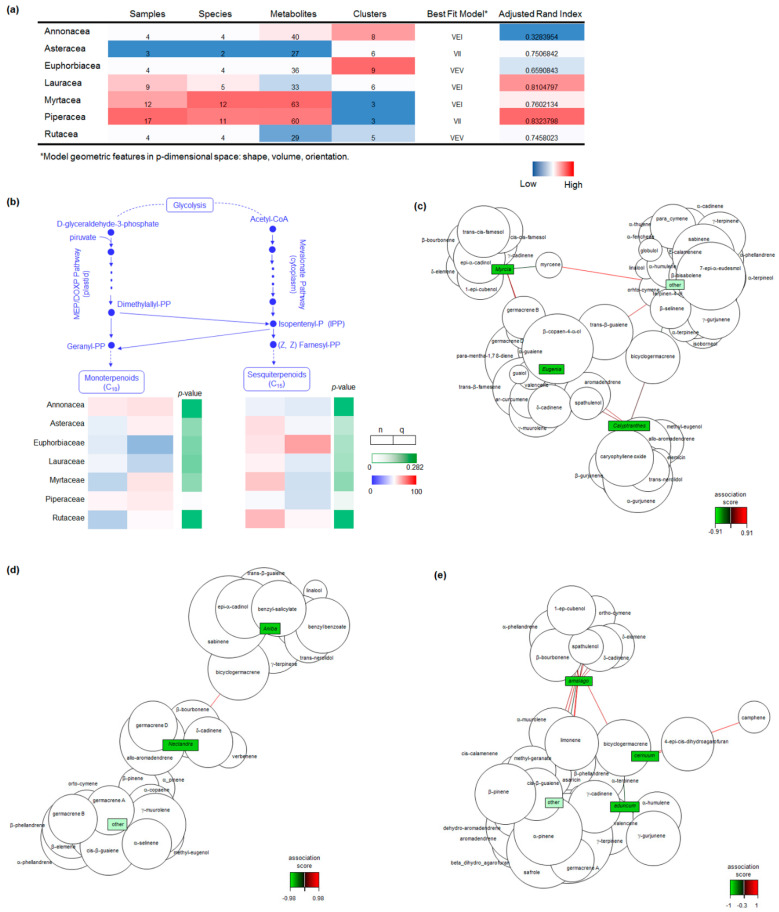
Chemical diversity of the EO composition within the most frequent botanical families. (**a**) Heatmap summary of GMMs of the EO chemical profiles of the most frequent botanical families. (**b**) Contribution of MT and ST metabolites to the qualitative (n) and quantitative (q) EO profile in the most frequently sampled aromatic families. Contribution is represented as a percentage of the composition in each botanical family. Relevance network for the identified metabolites in Myrtaceae (**c**) and Lauraceae (**d**) genera, and Piperaceae species (**e**). Association scores are represented as gradient for each network.

The significant intra-group variation prompted us to investigate the chemical diversity among the individuals within the most frequent botanical families, applying Gaussian Mixture (GMM) modelling to the EO chemical profiles (Figure 3 and Figure S6). The agreement between the model and the actual data classification, estimated by the adjusted Rand index (ARI), was higher than 65% for all botanical families, with the exception of Annonaceae (Figure 3). The best-fit GMM models demonstrated that quantitative differences in the chemical composition were the principal contributors to sample separation in Asteraceae and Piperaceae (model VII) and Myrtaceae and Lauraceae (model VEI), whereas, in Euphorbiaceae and Rutaceae (model VEV), qualitative chemical differences also contributed to the within-group covariance (Figure 3 and Figure S6). The groups of chemical profiles in the EOs from Euphorbiaceae were highly variable, although the number of identified metabolites was approximately 1.7-fold smaller than in Myrtaceae and Piperaceae (Figure 3 and Figure S6). The relative contribution of mono and sesquiterpenes to the best-fit GM model of the EOs’ composition was investigated and the qualitative (n) and quantitative (q) differences between the contribution of mono and sesquiterpenes to the EO models is shown (Figure 3). Monoterpenes had the most significant contribution to the composition of Piperaceae EOs, and sesquiterpenes to Myrtaceae, whereas benzyl alcohols are relevant metabolites in Lauraceae (Figure 3).

The metabolic models were associated with the phylogenetic classification at the genus-level for Lauraceae and Myrtaceae, and at the species-level for Piperaceae; these were the botanical families with the highest agreement between the theoretical model and the observed chemical composition. In Lauraceae, relevance networks demonstrated that benzyl alcohols were strongly associated with the genus *Aniba*, whereas allo-aromadendrene, germacrene D and δ-cadinene were more intricately linked to *Nectandra*. Myrcene, *trans*-β-guaiene and bicyclogermacrene were relevant to the EO composition of all of the investigated genera in Myrtaceae, although the relevance network analyses differentiated the chemical profile of EOs from the genera *Myrcia*, *Eugenia* and *Calyptranthes* (Figure 3). Germancre B was relevant to *Myrcia* and *Eugenia*, whereas spathulenol and aromadendrene were significant to *Eugenia* and *Calyptranthes* (Figure 3). In Piperaceae, the metabolic profile of *Piper amalago* EOs was the most divergent, with contributions from α-phellandrene, β-bourbonene and 1-epi-cubenol, whereas several metabolites were shared with *P. aduncum* and *P. cernuum* EOs, although high camphene levels and the presence of dihydro-agarofuran sesquiterpenes were exclusive to the latter (Figure 3).

Simultaneous hierarchical clustering of the samples based on the PLS similarity matrix and relevance network analyses demonstrated that the significant associations were caused by the presence or absence of specific metabolites, such as dihydro-agarofuran sesquiterpenes in *Piper cernuum* and benzyl alcohols in Lauraceae, and the absence of monoterpenes in Salicaceae and Sapindaceae (Figure 1, Figure 2 and Figure 3). Among the distinct metabolite profiles, monoterpenes were not detected in Eos from *Casearea sylvestris* (Salicaceae) and *Cupania vernalis* (Sapindaceae).

### 2.2. Chemical Composition and Antimicrobial Activity

**Figure 4:**
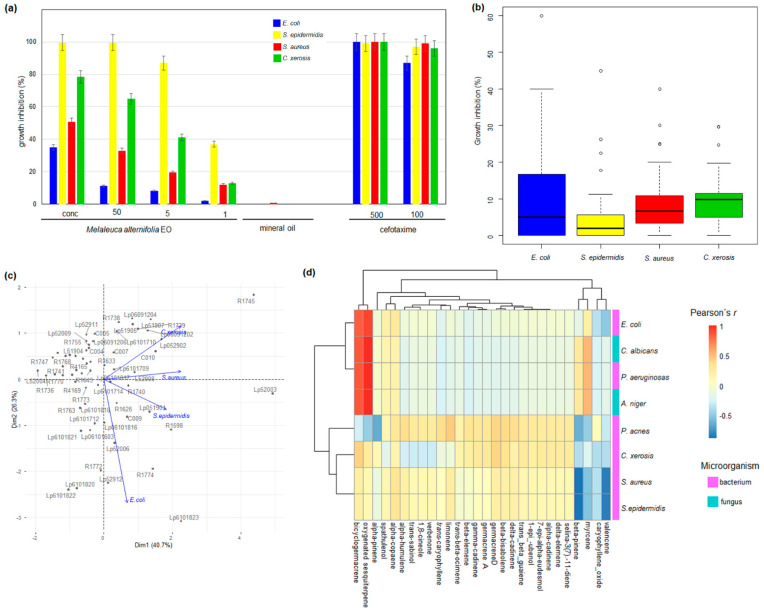
Biological activity of the EOs against pathogenic bacteria. (**a**) Dose-response estimation with positive and negative controls. (**b**) Growth inhibition amplitude for *E. coli*, *S. aureus*, *S. epidermidis* and *C. xerosis*. (**c**) PCA biplot of bacterial growth inhibition by the individual EOs. (**d**) Heatmap representation of the correlation between EO composition and minimum inhibitory concentration for pathogenic fungi and bacteria. Pearson’s correlation coefficients and their corresponding *p*-values are shown in Table S2.

Certified tea tree (*Melaleuca alternifolia* (Maiden and Betche) Cheel) EO and four pathogenic bacteria were used to determine the working concentration for the growth inhibition assays (Figure 4). The dilution medium (mineral oil) did not interfere with bacterial growth, whereas, the broad-spectrum antibiotics (cefotaxime) prevented bacterial growth at 100 µg.mL^−1^ (Figure 4). Concentrated EO completely inhibited *Staphylococcus epidermidis* growth and caused 35%, 51%, and 78% reduction in the propagation of *Escherichia coli*, *S. aureus*, and *Corynebacterium xerosis*, respectively (Figure 4). The growth inhibition of the frequent skin and mucous membrane colonizers *S. epidermidis* and *C. xerosis* were the most responsive to EO treatment (Figure 4), whereas *E. coli* and *S. aureus* were less affected by EO-induced growth inhibition (Figure 4). The tea tree EO concentration at 5% (*v*/*v*) allowed us to clearly identify growth inhibition for the investigated pathogens and was employed in the large-scale disk diffusion agar assays of the antimicrobial potential of the EOs from the rainforest species.

Most of the EOs from the rainforest plants exhibited antibacterial activity, although individual EO antibacterial activity was highly variable (Figure 4 and Figure S7). The maximum growth inhibition reached up to 60% against *E. coli* (Lp6101823, from *Helietta apiculata*, Rutaceae) (Figure 4 and Figure S7). Other EOs also exhibited a high potential to impair bacterial growth, such as 45% against *S. epidermidis* (R1598, *Guatteria australis*, Annonaceae), 40% against *S. aureus* (R1745, *Calyptranthes lanceolata*, Myrtaceae) and 30% against *C. xerosis* (Lp52006, *Marlierea exocoriata*, Myrtaceae), representing 2.4-, 1.6-, 3.7- and 1.6-fold the inhibitory effect of certified *M. alternifolia* EO at the same concentration (Figure 4 and Figure S7). The composition of the bacteria cell wall influenced the susceptibility to EO activity, contributing to approximately 41% of the variation in the PC analyses (Figure 4). The weight of the Gram-negative type of cell wall (*E. coli*) most strongly affected the first component, whereas the Gram-positive wall assembly (staphylococci and *C. xerosis*) exerted greater influence on the first component (Figure 4). The capacity to inhibit bacterial growth by the EO from native rainforest species was often higher or equivalent to certified *Melaleuca alternifolia* oil (Figure S7).

Ten EOs were selected for Minimum Inhibitory Concentration (MIC) assays against the previously investigated bacteria, plus the opportunistic pathogen *Pseudomonas aeruginosas*, the skin pathogen *Propionibacterium acnes*, the filamentous fungus *Aspergillus niger*, and the infective yeast *Candida albicans* (Table 2). The antibacterial activity was confirmed for concentrations as low as 0.124 µL/mL (Lp6101712, *Xylopia aromatica*, Annonaceae) against *C. xerosis*. *Aspergillus niger* and *Candida albicans* growth was impaired by all tested EOs at 0.5 µL/mL (Table 2). The correlation between the contents of the major metabolites in the essential oils and their antibacterial activity was investigated and are represented as a heatmap (Figure 4), and the statistical significance is presented in Table S2. The contents of oxygenated sesquiterpenes and bicyclogermacrene were positively correlated with the inhibition of *E. coli*, *C. albicans*, *P. aeruginosa*, and *A. niger* (Figure 4, Table S2). The myrcene contents were also positively correlated with the impairment of *C. albicans*, *P. aeruginosa*, and *A. niger* propagation, whereas the levels of limonene were positively correlated with the inhibition of *P. acnes* (Figure 4, Table S2). The contents of several major metabolites exhibited weak correlation with the antimicrobial activity against the tested pathogens (Figure 4).

**Figure 5:**
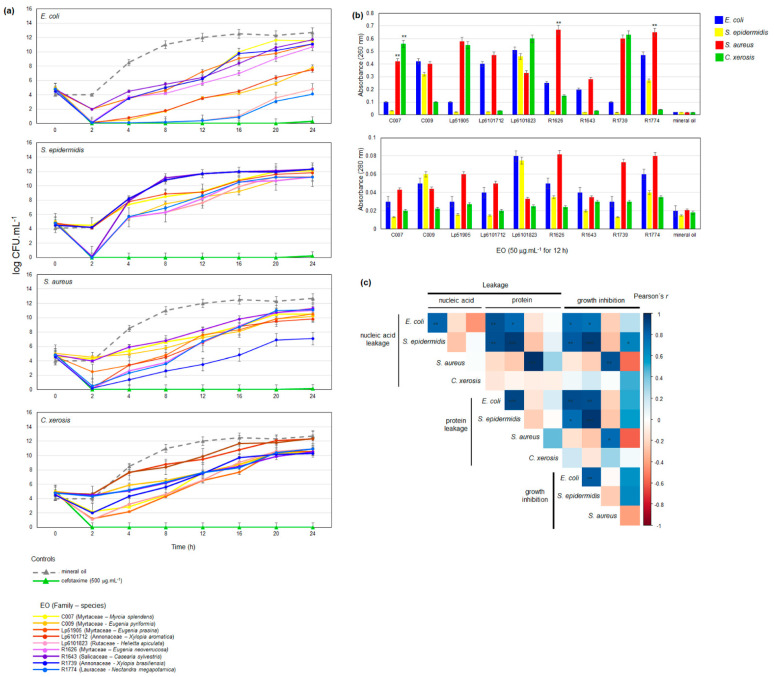
Eos antibacterial activity mode of action investigation. (**a**) Time-kill kinetics against pathogenic bacteria. Positive and negative control curves are represented by triangle and EO by circle markers and colors. (**b**) Cell component leakage assay, for nucleic acid (absorbance at 260 nm) and protein (absorbance at 280 nm). (**c**) Heatmap representation of the correlation between cell component leakage and inhibition of bacterial growth. Significance levels are represented as: ‘***’ 0.001, ‘**’ 0.01, and ‘*’ 0.1. Pearson’s correlation coefficients and their corresponding *p*-values are shown in Table S3.

To investigate the mechanism of action of the EOs against the tested pathogenic bacteria, we employed time-kill and cell component leakage assays (Figure 5). The bacterial kinetics of the EOs demonstrated that complete killing was reached 2 h after treatment at MIC with the EOs from Myrtaceae and Annonaceae against *E. coli*, Rutaceae, Myrtaceae, Salicaceae, Annonaceae, and Lauraceae against *S. epidermidis* and *S. aureus* (Figure 5). None of the tested EOs were able to induce the complete killing of *C. xerosis* at MIC, although most of them were able to reduce propagation up to 8 h after treatment (Figure 5). The investigated EOs induced cell component leakage at MIC for all of the tested bacterial species (Figure 5). The loss of nucleic acid and protein was detected, suggesting that EO treatment caused the formation of non-selective pores. The investigated EOs caused a greater loss of intracellular nucleic acids to *S. aureus* and *C. xerosis*, whereas protein leakage was higher in *E. coli*, *S. aureus*, and *C. xerosis* (Figure 5). As shown in the growth inhibition, MIC and time-kill assays, the most effective EOs for inducing bacterial intracellular component losses were from *Helietta apiculata* (Rutaceae, Lp6101823), *Xylopia brasiliensis* (Annonaceae, R1739), and *Nectandra megapotamica* (Lauraceae, R1774) (Figure 5). Correlation analyses demonstrated that growth inhibition was positively correlated to nucleic acid leakage for *E. coli* and, to a lesser extent, *S. epidermidis* (Figure 5, Table S3). In contrast, for *S. aureus*, growth impairment was positively associated with protein loss (Figure 5, Table S3).

**Figure 6:**
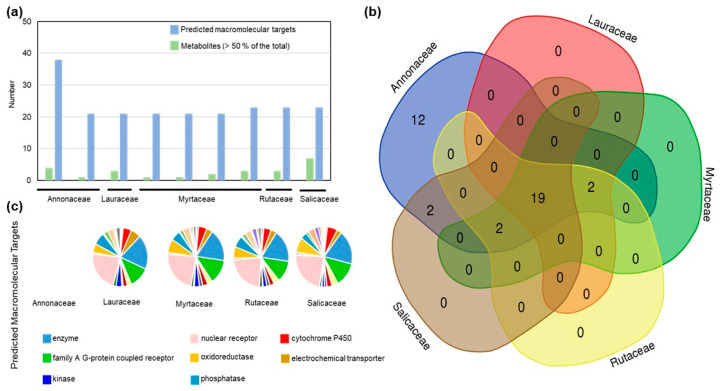
Pharmacokinetics properties of the EOs. (**a**) Number of metabolites and predicted macromolecular targets in the EOs per botanical family. Venn diagram (**b**) and pie chart (**c**) of the predicted ADME class targets of the metabolites in the EOs per botanical family. The complete list of predicted targets aggregated for each botanical family is shown in Table S5.

The performance of the EOs from the rainforest plants against pathogenic microorganisms prompted us to investigate the pharmacokinetic properties of their major components using in silico tools (Figure 6). The Absorption, Distribution, Metabolism, and Excretion (ADME) properties, such as the number of heavy atoms, number of aromatic heavy atoms, fraction Csp3, number of rotatable bonds, H-bond acceptors and donors, molecule predicted solubility, absorption, CYP inhibition prediction, violation of Lipinski, Ghose, Veber, Egan, and Muegge parameters, bioavailability score, PAINS and Brenk alerts, Lead-likeness violations and predicted synthetic accessibility, were investigated for 27 major EO components (Table S4). Most of the metabolites present in the EOs exhibited adequate drug-like predicted properties, individually (Table S4), indicating their medicinal potential alone or in combination with other metabolites found in EOs. The complexity of EO composition was not reflected in the number of predicted macromolecular targets, as EOs with a greater number of major metabolites, such as R1643 from *Casearia sylvestris* (Salicaceae), exhibited a similar number of predicted targets than those with a simpler composition, such as Lp6101712, from *Xylopia aromatica* (Annonaceae), where limonene represented more than 71% of the EO metabolites (Table 1, Figure 6). The EOs from Annonaceae (R1739 and Lp6101712, from *Xylopia brasiliensis* and *X. aromatica*) had the most divergent number of predicted macromolecular targets, with 12 exclusive categories including the classes Eraser, Primary active transporter, Other nuclear protein, Lyase, Reader, Transferase, and Ligase (Figure 6, Table S5). The majority of the EO metabolites displayed the predicted macromolecular targets of pharmacological interest (Figure 6).

## 3. Discussion

Essential oils are among the most studied plant extracts for treating infectious diseases and controlling microbial growth, primarily due to the antimicrobial activity of terpenes, phenylpropanoids, and flavonoids [3,8,10,13]. The antimicrobial mechanisms and molecular target sites of the metabolites in plant EOs are distinct from those of traditional antimicrobial agents, making them important elements in combinatorial strategies against infectious microorganisms [10,13]. To address the knowledge gap in the antimicrobial potential of EOs from the highly diverse rainforest, we have botanically classified and chemically characterized the EOs of plant species from nine areas. The isolated EOS were further characterized for antimicrobial and biocidal activity through agar diffusion assays, minimum inhibitory concentration, time-kill, and cell-component leakage assays. Subsequently, we investigated the pharmacokinetic properties of the EO compounds using in silico tools.

The chemical profiling of the EOs confirmed the roles of inter- and intra-specific genetic variation and environmental conditions in determining the metabolic diversity of rainforest plants [12]. In the EOs from 50 species, we identified 113 distinct metabolites. In contrast, the chemical characterization of the EOs from 48 Lamiaceae species, including basil, rosemary, lavender, and peppermint, revealed 83 compounds [14], although a review work demonstrated that 150 compounds have been identified in EOs from *Rosmarinus officinalis* L. alone [15]. In two commercial cultivars of lavender and lavandin, the chemical characterization revealed 50 compounds in the EOs [16]. Fruits from the native African *Xylopia aethiopica* produced EOs with 14 identified metabolites in GC-MS analyses [17]. Employing high-speed countercurrent GC, 15 compounds were identified in the EOs from the rainforest native *Piper mollicomum* [18,19]. The genus *Piper* is widely distributed throughout the tropics and more than 250 compounds were identified in the EOs from its species [20]. Thus, the resolution of the chemical profiles identified in our study are comparable to those reported for EOs from cultivated and wild aromatic plants. As observed in the cultivated and model plants [4,21,22], the investigated rainforest species also exhibited high intra-specific chemical variation, although family-specific compounds were also present, such as geraniol in Annonaceae and η-octane in Euphorbiaceae. Among the distinct metabolite profiles, monoterpenes were not detected in the EOs from *Casearea sylvestris* (Salicacea) and *Cupania vernalis* (Sapindaceae). Although known as a sesquiterpene-rich species, monoterpenes have been identified in the Eos of *C. sylvestris* [23,24]. The metabolite profile of *C. vernalis* remains poorly characterized, although several extracts were demonstrated to exhibit biological activity [25]. The significant intra-group variation in their EO chemical composition was further investigated by applying Gaussian Mixture (GM) modelling. GM clustering has a probabilistic nature and does not assume independence between adjacent measures, making it suitable to study metabolites synthesized by the same or shared pathways [26,27]. The chemical profiles of the EOs from Euphorbiacea were highly variable, mostly due to quantitative differences [28,29], as shown for the *Croton* species. The relative contribution of the chemical classes to EO composition was variable, with a predominance of monoterpenes in Piperaceae, sesquiterpenes in Myrtaceae, and benzyl alcohols in Lauraceae. The principal biosynthetic pathway of monoterpenes in plants is the MEP/DOPX localized in plastids, whereas sesquiterpenes are synthesized from precursors of the mevalonate pathway in the cytosol, although interaction between the pathways are known [6,30]. The scaffold of sesquiterpenes in plants is catalyzed by Terpene Synthases (TPS), which produce structurally distinct acyclic, mono-, bi- and tri-cyclic ST from common prenyl diphosphate precursors [31]. Genomic studies have associated the transcription of TPS genes to the ST profile in Myrtaceae [32,33], Lauraceae [34,35], and Piperaceae [36]. The shikimate pathway and phenylalanine biosynthesis from chorismate produce volatile benzenoids, although the information is from plant reproductive structures and does not include Lauraceae [37]. The extensive intra-specific chemical diversity of aromatic and medicinal plant species is challenging for several steps required for their widespread use, including yield, cultivation, and extraction conditions [38]. Similarly, the chemical variation within a given species reinforces the need for genetic and chemical profiling of the individuals of interest [39,40].

The antibacterial activity of the isolated EOs was initially investigated by agar diffusion assays against the frequent skin and mucous membrane colonizers *Staphylococcus epidermidis* and *Corynebacterium xerosis* [41,42], as well as the leading bacterial pathogens in healthcare-associated infections, *Escherichia coli* and *S. aureus* [43]. *S. aureus*, along with *Enterococcus* spp., *Klebsiella* spp., *Acinetobacter baumannii*, *Pseudomonas aeruginosa*, and *Enterobacter* spp., constitutes the “ESKAPE” group of multi-drug resistant pathogens [8]. Antibacterial activity was observed for most of the EOs from the rainforest, at higher levels than that observed for certified *Melaleuca alternifolia* oil. The composition of the bacterial cell wall determined the susceptibility to EO activity, as shown previously [8,13,44]. The antimicrobial activity of the EOs was more variable for the Gram-negative type of cell wall (*E. coli*), whereas the Gram-positive wall assembly (staphylococci and *C. xerosis*), consisting of glycopolymers and proteins, associated with teichoic acids, polysaccharides, and proteins, was less affected by the investigated EOs. These observations agree with the roles of EO compounds in the destabilization of bacterial cellular architecture, due to the disruption of the membrane’s integrity, leading to the impairment of cellular activities, such as energy production and membrane transport, and the loss of cellular components and ions [44]. These observations suggest a potential harmful effect of the EOs to non-target cells; however, their use in cosmetics and household products has been shown to be safe and able to impair bacterial growth. Moreover, combinatory therapeutic alternatives and topic applications may contribute to reducing mutagenic, cyto- and geno-toxic, effects.

The metabolite profile and synergistic interactions among the compounds are critical to EO antimicrobial activity [9,44,45]. The reactivity of the metabolites is associated with their antimicrobial potential, as oxygenate and cyclic molecules have higher inhibitory effect on microorganisms than hydrocarbons in mixtures or isolated [9,44]. The interactions among the EO metabolites responsible for antimicrobial properties may lead to the enhanced activity or attenuation of negative effects [44,45]. Moreover, the distinct molecular structure of the EO compounds allow them to display a broader spectrum of action in comparison to isolated substances [44,45].

The ability to inhibit bacterial growth allowed us to choose ten EOs for the MIC assays, using the “ESKAPE” opportunistic pathogen *Pseudomonas aeruginosas*, the skin pathogen *Propionibacterium acnes*, in addition to the previously investigated bacteria, along with the filamentous fungus *Aspergillus niger* and the infectious yeast *Candida albicans*. The MIC assays confirmed the antimicrobial potential of the EOs, at levels similar to those reported for oils from commercially cultivated plants, such as rosemary and thyme [8,46]. The contents of oxygenated sesquiterpenes, bicyclogermacrene and myrcene were positively correlated to microbial growth inhibition. Recent studies have demonstrated that phenolic terpenoids display higher antibacterial activity against Gram-negative and Gram-positive bacteria [47]. The author observed that treatment with phenolic terpenoids carvacrol and thymol immediately caused the loss of cell membrane integrity and ion leakage. The importance of the hydroxyl group of the phenol moiety was also highlighted in the work, as O-methyl derivatives and benzylic partners were shown to be ineffective [47]. These observations agree with the correlation results in our study. However, the contents of several major metabolites exhibit weak correlation with the antimicrobial activity against the tested pathogens, indicating a synergistic effect among the EO metabolites [8,48]. The combination of carvacrol, thymol, eugenol and nootkatone was shown to exert the bacteriostatic and bactericidal effects, even at low concentrations, highlighting the complementary effect of the different compounds in the EO [48].

The mechanism of the EOs’ action against the pathogenic bacteria was investigated by time-kill and cell component leakage assays. The kill kinetics assays confirmed fast bactericidal action, as shown previously for EOs from cultivated *Origanum vulgare* [49,50,51] and tea tree [52]. However, viable cells were detectable at later stages for all treatments, including broad-spectrum commercial antibiotics. Treatment with the EOs at MIC induced the loss of nucleic acid and protein, suggesting the formation of non-selective pores. The time-kill and cell component leakage results agree with the proposed modes of action of EOs against bacterial pathogens, functioning to destabilize the cell structure, then leading to perturbations in the integrity of the membrane system, disrupting several cellular activities, such as energy production and cellular transport [44,51,52]. The disruption of bacterial membranes by EOs appears to be non-selective and to induce a general leakage of the cellular components and the loss of ions [44], although our indirect evidence from correlation analyses indicate that EO-induced nucleic acid leakage is more prejudicial to *E. coli* and *S. epidermidis* than to *S. aureus* and *C. xerosis*.

The use of plant-derived compounds in pharmaceutical applications is dependent on their pharmacokinetic properties, Absorption, Distribution, Metabolism, and Excretion (ADME), which is, in turn, dependent on the chemical structure of its individual components [38,45]. In silico predictive tools demonstrated that the individual metabolites found in the investigated EOs exhibit adequate drug-like predicted properties. Moreover, the predicted macromolecular targets of the individual metabolites include several classes of pharmacological interest, such as kinases, phosphatases, nuclear receptors and cytochrome P450. The complexity of EO composition did not reflect the number of predicted macromolecular targets, but was associated with the molecular structure of its metabolites. The EOs isolated from *Xylopia brasiliensis* and *X. aromatica* (Annonaceae) were predicted to have 12 exclusive classes of macromolecular targets. The identification of macromolecular targets of pharmacological interest suggests that the EOs may have further applications in drug composition.

## 4. Materials and Methods

### 4.1. Biological Samples Collection and Environmental Data

Aromatic plants were sampled from nine Atlantic rainforest reserves at experimental stations managed by Agência Paulista de Tecnologia dos Agronegócios (APTA) (Figure S1) for botanical identification, herbarium mounts and chemical analyses. The families of aromatic plants were selected based on their reported biological activity, aroma emission and plant distribution (Table S6).

Plants were tagged and the coordinate reference determined by Global Positioning System (GPS). Voucher specimens were deposited at the Herbarium of Instituto Agronômico (IAC) (http://herbario.iac.sp.gov.br/ (accessed on 7 December 2022)), under the given accession numbers (Table 1) and classified according to the list of species of Brazilian flora [53].

### 4.2. Essential Oil Extraction

As this was a study of native aromatic plants, in order to preserve the species, only the vegetative aerial parts were sampled, and the essential oils were extracted exclusively from the leaves. The leaves were detached from the stalks and air-dried at room temperature, in the absence of direct light. The EOs were extracted from 54 to 1870 g of dry material, depending on availability, for two hours, by hydrodistillation in Clevenger-type apparatus, according to the Brazilian Pharmacopeia [54]. The oils were stored in hermetically closed vials at −20 °C before chemical profiling. Yield is represented as oil weight (g) per dry material weight (g).

### 4.3. Chemical Characterization and Quantification of Essential Oils

The chemical composition of the EOs was determined by gas chromatography coupled with mass spectrometry (GC-MS Shimadzu, model QP-5000, Kyoto, Japan), equipped with fused silica capillary column OV—5 (30 m × 0.25 mm × 0.25 μm, Ohio Valley Specialty Chemical, Inc., Marietta, OH, USA), using Helium as a carrier gas (1.0 mL min^−1^); operating with injector temperature of 220 °C, the transfer line was kept at 230 °C, a split ratio of 1:20, and an injection volume of 1.0 µL of EO solution (1 µL essential oil/1 mL ethyl-acetate, chromatography grade) was employed using the auto-sampler. The GC was operated under temperature-programmed conditions, between 60 °C and 240 °C, by 3 °C per min^−1^. The MS data were acquired in the full-scan mode (*m/z* 40–450) using the electron ionization (EI), with an ionization voltage of 70 eV. The quantitative analyses were performed by the area normalization method, as triplicate readings, by gas chromatography with a flame ionization detector (GC–FID Shimadzu, model GC-2010). The analyses were conducted under the same oven operating conditions used in GC-MS. The metabolites were identified by the comparative analyses of mass spectra against the system database (Nist 62.lib) and by retention indices [55] obtained from the injection of a mixture of *n*-alkanes (C_9_H_20_-C_25_H_52_, Sigma Aldrich, St. Louis, MO, USA, 99%), applying the equation described by Van den Dool and Kratz [56]. The metabolites were considered as major components when representing ≥ 10%. The complete chemical data and their associated metadata are deposited at the National Metabolomics Repository (https://www.metabolomicsworkbench.org/data/index.php (accessed on 7 December 2022)), under identifier ST000606.

### 4.4. Microbial Strains

Certified cultures of *Escherichia coli* (ATCC 8739), *Staphylococcus aureus* (ATCC 6538), *S. epidermidis* (ATCC 12228), *Corynebacterium xerosis* (ATCC 373), *Pseudomonas aeruginosa* (ATCC 9027), *Propionibacterium acnes* (ATCC 11827), *Candida albicans* (ATCC 10231) and *Aspergillus niger* (ATCC 16404) were provided by Instituto Adolfo Lutz (São Paulo, SP, Brazil). Bacterial cultures were started from isolated colonies, and fungal cultures, from single spores. The bacterial concentration was estimated based on spectrophotometric absorbance readings at 600 nm for *E. coli* and *P. aeruginosa*, 490 nm for *S. epidermidis* and *P. aeruginosa*, 530 nm for *S. aureus* and 578 nm for *C. xerosis*, for McFarland turbidity standard.

### 4.5. Estimation of EO Effective Concentration for Antimicrobial Activity

Certified commercial essential oil from tea tree (*Melaleuca alternifolia*, (Maiden and Betche) Cheel) was used to estimate the effective concentration for antimicrobial activity analyses. Serial EO dilutions were prepared in sterile mineral oil in a volume/volume basis up to a total of 200 µL. The bacterial cultures were diluted to 0.5 McFarland standards in Tryptone Soy Broth (TSB, Oxoid Thermo Scientific, Loughborough, UK) liquid medium and supplemented with serial dilutions of tea tree EO. The suspensions were incubated at 37 ± 2 °C, with continuous agitation at 200 rpm for 36 h. Absorbance readings of 1 mL aliquots were used to calculate growth inhibition, in comparison to the negative control consisting of TSB supplemented with 200 µL of sterile mineral oil. Broad-spectrum antibiotics cefotaxime (Merck/Sigma-Aldrich, St. Louis, MO, USA) was used as the positive control at 500 µg mL^−1^. The minimum estimated concentration of tea tree EO impairing bacterial growth (5% *v*/*v*) was used in further analyses with the 63 EO samples from the rainforest plants.

### 4.6. Antimicrobial Activity Analyses

Antibacterial activity was investigated by growth inhibition in agar diffusion assays for four bacterial species, and minimum inhibitory concentration (MIC) analyses were used for the bacteria and fungi. For both assays, bacterial suspensions were initiated from inoculating a single colony to 20 mL of TSB (Oxoid ThermoScientific, UK) and grown to saturation at 28 °C, for approximately 14 h, with 200 rpm shaking. An aliquot of 1 mL was transferred to 20 mL of fresh medium, and the procedure was repeated twice. Bacterial concentrations on the final saturated suspension were corrected to 10^−8^ colony forming unit (CFU) per mL^−1^ by absorbance readings at 600 nm for *E. coli* and *P. aeruginosa*, 490 nm for *S. epidermidis*, 530 nm for *S. aureus*, and 578 nm for *C. xerosis*, to prepare the adjusted inocula. For the agar diffusion assays, 1 mL of the saturated bacterial culture was added to 400 mL of cooled, fused Nutrient Agar medium (Oxoid ThermoScientific, UK) and supplemented with 1.5 mL of a 2% (*w*/*v*) solution of 2,3,5-triphenyl tetrazolium chloride (TTC). The mixture was poured into 9 mm sterile Petri dishes containing five, evenly distributed, sterile aluminum rings with a diameter of 6 mm. The rings were removed from the solidified medium and 300 μL of essential oil at 5% (*w*/*v*) in sterile mineral oil were added to the wells. The plates were incubated horizontally in a bacteriological oven at 37 ± 2 °C for 48 h, and after growth, the abaxial surface of the plates was digitalized, and inhibition halos were measure in ImageJ2 [57], by applying the *Measure* function from the *Analyze* menu. *Melaleuca* EO (absolute and 5% (*w*/*v*)) and cefotaxime (500 µg mL^−1^) and the sterile mineral oils were used as positive and negative controls, respectively.

The minimal inhibitory concentration (MIC) was determined for nine EO samples using the broth microdilution method, according to CLSI guidelines [58]. The EO samples were diluted at 1% (*v*/*v*) in propylene glycol and submitted to serial dilutions (1:2) in sterile 96-well microplates containing 100 μL of TBS. Actively growing microorganisms from the adjusted cultures were diluted an optical density of 0.5 McFarland, equivalent to 2 × 10^6^ colony forming units (CFU).mL^−1^ in TSB and 20 μL of the diluted culture were added to the wells to give a final inoculum of approximately 1 × 10^5^ CFU.mL^−1^. The microplates were incubated at 37 ± 2 °C for 48 h for bacteria or at 25 ± 2 °C for 72 h for fungal and yeast cultures. The MIC values were determined by monitoring the microorganism growth at the adequate optical density in the presence of multiple concentrations of the EOs. After the incubation period, the plates were scanned for turbidity in an Enzyme Linked Immunosorbent Assay (ELISA) reader. The negative controls consisted of TS broth and TS broth inoculated with propylene glycol, without EO. The MIC values are presented as the smallest concentration inhibiting microorganism growth in µg of EO per mL ± standard error.

### 4.7. Mode of Action Investigations

The antimicrobial mode of action of the nine selected EO samples was investigated for bactericidal activity and cell-component leakage analyses, as described in the Clinical and Laboratory Standards Institute M26A approved guidelines [58]. In the time-kill kinetics analyses, bacterial suspensions were grown in the TSBup to mid logarithmic phase, as described. Dilutions corresponding to 0.5 McFarland (~10^−8^ CFU mL^−1^) were prepared in 10 mM PBS buffer at pH 7.4, and added to 20 mL TBS supplemented with 200 µL of EO at 50 µg mL^−1^. Aliquots were taken at 0, 2, 4, 8, 12, 16, 20 and 24 h after inoculation and plated on TBS agar in triplicate. The plates were incubated overnight at 37 °C and the bacterial colonies were counted. Cefotaxime and sterile mineral oil were used as positive and negative control, respectively.

The membrane permeability was investigated by cell-component leakage analyses, as described [59]. Bacterial suspensions at 0.5 McFarland turbidity (~10^8^ CFU mL^−1^) were supplemented with 50 µg.mL^−1^ of EO, incubated at 37 °C with continuous agitation at 200 rpm for 12 h. Intact bacterial cells were precipitated by centrifugation at 9000× *g* for 10 min at 4 °C and the contents of extracellular nucleic acids and proteins were determined by absorbance readings of the supernatant at 260 nm and 280 nm, respectively.

### 4.8. EO Metabolite Physicochemical and Pharmacokinetic Properties

The major metabolites of the nine selected EOs were compiled, their canonical Simplified Molecular-Input Line-Entry System (SMILES) format were obtained and their molecular structures were used for in silico prediction of physicochemical, drug-likeness, pharmacokinetics, medicinal chemistry friendliness, and Absorption, Distribution, Metabolism and Excretion (ADME) properties, using the SwissADME algorithm [60]. Macromolecular target prediction was carried out using SwissTargetPrediction algorithm [60].

### 4.9. Data Analyses

Data preprocessing and analyses were performed using R [61]. The oil yield and chemical composition data were averaged, centered and Pareto-scaled. Supervised and unsupervised multivariate and modelling analyses were performed using mixOmics [62] and mclust [63]. The best fitting model for the chemical data was determined by Bayesian Information Criterion (BIC) and Integrated Complete-data Likelihood (ICL) and include the number of mixing components and covariance parametrization [63]. For each component, several parameters were computed, including the mean and the variance, as well as the density mixing probabilities and the total number of gene pairs. Pearson coefficients and correlation significance levels were obtained in Hmisc [64] and represented graphically using corrplot [65].

This article does not contain any studies with human and/or animal participants performed by any of the authors. The sampling of native plants for research purposes is authorized under permit AD0077F, issued by Sistema Nacional de Gestão do Patrimônio Genético e do Conhecimento Tradicional Associado (SisGen).

The metabolomics and metadata reported in this paper are available at Metabolomics Workbench (https://www.metabolomicsworkbench.org/data/index.php (accessed on 7 December 2022)), study identifier ST000606.

## 5. Conclusions

In the current study, we have investigated the antimicrobial potential of the EOs from aromatic plants from the Atlantic rainforest. EOs were isolated from 63 plants, comprising 15 botanical families. The EOs’ chemical compositions consisted of 113 distinct metabolites, primarily mono and sesquiterpenes. Multivariate analyses detected extensive inter- and intra-specific variation in the chemical profiles of the EOs. These observations were confirmed by Gaussian models, which revealed distinct contributions of quantitative and qualitative differences within the botanical families. Relevance networks allowed the identification of genera-specific metabolites for Lauraceae and Myrtaceae, and species-specific profiles for Piperaceae. The EOs exhibited extensive antimicrobial potential against pathogenic bacteria and fungi, and the biocidal capacity was demonstrated for a selected group of EOs. The EOs’ treatment of pathogenic bacteria promoted a fast reduction in the number of viable, colony-forming cells, and caused the loss of cellular components. In silico analyses demonstrated that the major metabolites in the EOs have adequate pharmacokinetic properties and interesting predicted pharmacological targets. Our results may contribute to the development of new plant-based antimicrobial products.

## Figures and Tables

**Table 1:** Main components (≥10%) of the essential oil extracted from plant species from the Atlantic rainforest. Plant species are presented within botanical families. Latitude and longitude coordinates are represented as decimal values. Essential oil yield is presented as dry weight (*w*/*w*) percentages. Complete chemical profile, literature, and calculated retention indices (RI) and experiment metadata are deposited at the National Metabolomics Repository under identifier ST000606.

Family/Species	Sample/Herbarium Code	Location (Coordinates, Elevation)	Yield (%)	Major Components (%)
Anacardiaceae				
*Schinus terebinthifolius* Raddi	R1772/IAC 47521	Ribeirão Preto (47°51′58.72″ S, 21°12′52.36″ W, 570 m)	0.26	α-phellandrene (23.2); α-pinene (18.2); β-phellandrene (16.8)
Annonaceae				
*Annona dioica* A.St.-Hil.	Lp06101603/IAC 47955	Votuporanga (50°3′55.60″ S, 20°27′41.20″ W, 463 m)	0.37	bicyclogermacrene (30.1); germacrene D (21.0); *trans*-caryophyllene (12.2)
*Guatteria australis* A.St.-Hil.	R1598/ IAC 46831	Ubatuba (45°7′39.30″ S, 23°25′18.60″ W, 26 m)	0.14	spathulenol (27.4); caryophyllene oxide (18.8)
*Xylopia aromatica* (Lam.) Mart.	Lp6101712/ IAC 47969	Votuporanga (50°3′34.99″ S, 20°27′15.20″ W, 4 m)	0.16	limonene (71.7)
*Xylopia brasiliensis* Spreng.	R1739/IAC 47266	Pariquera-Açu (47°52′48.76″ S, 24°36′48.42″ W, 25 m)	0.17	1,8-cineole (11.1); spathulenol (28.3)
Araceae				
*Monstera* cf. *adansonii* Schott	L52008/IAC 47079	Ubatuba (45°7′47.57″ S, 23°24′49.97″ W, 180 m)	0.14	β-phellandrene (36.7), α-pinene (17.2), 2 tridecanone (17.0)
Araliaceae				
*Dendropanax cuneatus* (DC.) Decne. & Planch.	C010/IAC 47099	Jundiai (46°55′40.69″ S, 23°6′42.70″ W, 770 m)	0.12	caryophyllene oxide (15.6), trans caryophyllene (13.2), β pinene (10.9)
*Dendropanax cuneatus* (DC.) Decne. & Planch.	Lp6101818/IAC 47975	Adamantina (51°9′6.80″ S, 21°39′47.01″ W, 380 m)	0.004	spathulenol (22.1), trans caryophyllene (18.4), bicyclogermacrene (15.7), δ-3-carene (12.5)
*Dendropanax cuneatus* (DC.) Decne. & Planch.	R1770/IAC 47519	Mococa (46°59′55.30″ S, 21°25′24.56″ W, 568 m)	0.21	bicyclogermacrene (32.8)
Asteraceae				
*Baccharis dracunculifolia* DC.	R1755/IAC 47282	Pariquera-Açu (46°59′44.79″ S, 24°37′15.32″ W, 25 m)	0.42	*trans*-nerolidol (30.5), β-copaen-4-α-ol (12.0), limonene (11.6)
*Baccharis dracunculifolia* DC.	R1773/IAC 47522	Ribeirão Preto (47°52′12.64″ S, 21°11′27.63″ W, 557 m)	0.54	*trans*-nerolidol (27.3), limonene (17.4)
*Cyrtocymura scorpioides* (Lam.) H. Rob.	C005/IAC 47097	Jundiai (46°55′40.69″ S, 23°6′42.70″ W, 770 m)	0.32	germacrene D (36.1), β-pinene (26.6)
Euphorbiacae				
*Croton celtidifolius* Baill.	Lp52912/IAC 29030	Monte Alegre do Sul (46°40′30.18″ S, 22°41′58.52″ W, 743 m)	0.16	cis-β-guaiene (15.8), germacrene D (11.7), *trans*-nerolidol (11.1)
*Croton floribundus* Spreng.	R4167/IAC 46976	Campinas (47°4′3.36″ S, 22°51′45.72″ W, 670 m)	0.09	*trans*-caryophyllene (21.9), caryophyllene oxide (13.7)
*Croton urucurana* Baill.	R1768/IAC 47517	Mococa (46°59′55.30″ S, 21°25′24.56″ W, 568 m)	0.11	bicyclogermagrene (43.4), germacrene D (24.0)
*Croton warmingii* Müll. Arg.	R4165/IAC 46974	Campinas (47°4′3.57″ S, 22°51′45.79″ W, 670 m)	0.15	bicyclogermacrene (17.4), *trans*-caryophyllene (16.8)
Lauraceae				
*Aiouea* sp.	R1736/IAC 47263	Pariquera-Açu (46°59′44.79″ S, 24°37′15.32″ W, 25 m)	0.28	α-phellandrene (24.5), *trans*-nerolidol (19.4)
*Aniba viridis* Mez	R1746/IAC 47273	Pariquera-Açu (47°52′48.76″ S, 24°36′48.42″ W, 25 m)	0.59	benzyl salicylate (23.4), benzyl benzoate (14.1)
*Aniba viridis* Mez	Lp51907/ AC 47071	Ubatuba (45°7′39.29″ S, 23°25′18.59″ W, 29 m)	0.42	linalool (11.1), *trans*-nerolidol (73.1)
*Endlicheria paniculata* (Spreng.) J.F.Macbr.	R1622/IAC 46801	Campinas (47°4′3.30″ S, 22°51′50.00″ W, 652 m)	0.05	α-selinene (34.5), spathulenol (15.3), γ-muurolene (11.8)
*Nectandra megapotamica* (Spreng.) Mez	Lp6101820/IAC 47986	Adamantina (51°9′7.50″ S, 21°39′47.00″ W, 349 m)	0.13	*cis*-β-guaiene (23.4), spathulenol (15.6)
*Nectandra megapotamica* (Spreng.) Mez	R1761/IAC 47510	Mococa (46°58′51.65″ S, 21°26′53.71″ W, 600 m)	0.27	α-pinene (27.1), β-pinene (28.2), bicyclogermagrene (16.4)
*Nectandra megapotamica* (Spreng.) Mez	R1774/IAC 47523	Ribeirão Preto (47°52′12.64″ S, 21°11′27.63″ W, 557 m)	0.10	oxygenated sesquiterpene (28.1), α-pinene (18.7), β-pinene (17.3)
*Nectranda megapotamica* (Spreng.) Mez	Lp052902/IAC 47084	Monte Alegre do Sul (46°39′57.60″ S, 22°42′15.12″ W, 778 m)	0.07	*cis*-β-guaiene (22.7), α-pinene (21.2), β-pinene (18.5)
*Ocotea odorifera* (Vell.) Rohwer	R1747/IAC 47274	Pariquera-Açu (47°52′48.76″ S, 24°36′48.42″ W, 25 m)	2.88	camphor (50.5), methyl-eugenol (20.0)
Meliaceae				
*Trichilia elegans* A.Juss.	Lp6101709/IAC 47961	Votuporanga (50°3′30.60″ S, 20°27′27.50″ W, 479 m)	0.28	germacrene B (44.3)
Myristicaceae				
*Virola bicuhyba* (Schott ex Spreng.) Warb.	Lp52003/IAC 49465	Ubatuba (45°7′20.86″ S, 23°24′34.06″ W, 50 m)	0.14	*cis*-β-guaiene (21.4), *trans*-caryophyllene (18.1)
Myrtaceae				
*Calyptranthes lanceolata* O.Berg	R1745/IAC 47272	Pariquera-Açu (47°52′48.76″ S, 24°36′48.42″ W, 25 m)	0.12	methyl eugenol (80.4)
*Calyptranthes lucida* Mart. ex DC.	Lp52009/IAC 47080	Ubatuba (45°7′39.29″ S, 23°25′18.59″ W, 30 m)	0.19	caryophyllene oxide (17.3), *trans*-caryophyllene (16.9), bicyclogermacrene (12.4)
*Campomanesia guavirota* (DC.) Kiaersk.	Lp6101822/IAC 47988	Adamantina (51°9′8.20″ S, 21°39′46.50″ W, 355 m)	0.33	α-pinene (12.3), linalool (11.8)
*Eugenia moraviana* O.Berg.	Lp06101816/ IAC 47973	Adamantina (51°9′6.19″ S, 21°39′47.80″ W, 373 m)	0.04	β-pinene (16.2), *trans*-caryophyllene (14.2), β elemene (11.0)
*Eugenia neoverrucosa* Sobral	R1626/IAC 46825	Campinas (47°4′0.30″ S, 22°51′52.03″ W, 650 m)	0.42	α-pinene (94.5)
*Eugenia prasina* O.Berg	Lp51905/IAC 47069	Ubatuba (45°7′39.29″ S, 23°25′18.59″ W, 29 m)	0.28	limonene (61.4), α-pinene (12.6)
*Eugenia pyriformis* Cambess.	C009/IAC 34660	Jundiai (46°55′40.69″ S, 23°6′42.69″ W, 770 m)	0.17	β-pinene (39.7), α-pinene (31.5)
*Eugenia myrcianthes* Nied.	Lp6101714/ IAC 47971	Votuporanga (50°3′30.10″ S, 20°27′20.99″ W, 488 m)	0.06	β-copaen-4-α-ol (31.7)
*Marlierea exocoriata* Mart.	Lp52006/IAC 47077	Ubatuba (45°7′8.70″ S, 23°24′32.52″ W, 38 m)	0.28	α-pinene (37.6), β-pinene (18.2), sabinene (11.2)
*Myrcia spectabilis* DC.	Lp051901/IAC 47045	Ubatuba (45°7′39.29″ S, 23°25′18.59″ W, 29 m)	0.41	*trans*-*cis*-farnesol (52.1), *cis*-*cis*-farnesol (41.1)
*Myrcia splendens* (Sw.) DC.	C007/IAC 37365	Jundiai (46°55′40.51″ S, 23°6′42.52″ W, 770 m)	0.21	α-pinene (28.1), germacrene D (20.9)
*Myrcia tomentosa* (Aubl.) DC.	Lp06101817/IAC 47974	Adamantina (51°9′6.40″ S, 21°39′46.70″ W, 370 m)	0.15	germacrene D (33.09%), *trans*-caryophyllene (20.41%),
Piperaceae				
*Piper aduncum* L.	Lp52911/IAC 47090	Monte Alegre do Sul (46°40′20.99″ S, 22°42′0.36″ W, 743 m)	0.51	spathulenol (10.6), valencene (9.7), α-pinene (6.4), asaricin (14.9), safrole (13.3)
*Piper aduncum* L.	Lp6101606/IAC 47958	Votuporanga (50°3′53.10″ S, 20°27′46.30″ W, 458 m)	1.52	asaricin (80.1), safrole (10.8)
*Piper aduncum* L.	Lp6101608/IAC 47960	Votuporanga (50°3′53.10″ S, 20°27′46.60″ W, 465 m)	1.55	asaricin (73.4), safrole (10.5)
*Piper amalago* L.	Lp06091202/IAC 32056	Campinas (47°4′2.30″ S, 22°51′53.70″ W, 664 m)	0.20	β-phellandrene (39.3), α-pinene (14.8), germacrene D (11.7)
*Piper amalago* L.	Lp06091206/IAC 46823	Campinas (50°3′53.09″ S, 20°27′46.30″ W, 458 m)	0.36	β-phellandrene (15.9), α-pinene (6.7), sabinene (6.3), bicyclogermagrene (20.8), spathulenol (9.1)
*Piper amalago* L.	R1763/IAC 47512	Mococa (46°58′51.65″ S, 21°26′53.71″ W, 600 m)	0.26	β-phellandrene (33.1), α-pinene (11.7), bicyclogermagrene (15.0)
*Piper amalago* L.	Lp6101821/IAC 47987	Adamantina (51°9′7.89″ S, 21°39′47.19″ W, 349 m)	0.23	β-phellandrene (12.3), sabinene (8.2), myrcene (6.8), bicyclogermagrene (19.4); γ-muurolene (5.9), spathulenol (5.6)
*Piper amplum* Kunth.	R1740/IAC 7267	Pariquera-Açu (47°52′48.76″ S, 24°36′48.42″ W, 25 m)	0.38	α-pinene (18.1), *cis*-β-ocimene (10.5), limonene (8.6), *trans*-caryophyllene (8.8), germacrene D (5,5)
*Piper cernuum* Vell.	L51904/IAC 7068	Ubatuba (45°7′39.04″ S, 23°25′18.52″ W, 30 m)	0.32	α-pinene (10.0), camphene (6.3), dihydro-β-agarofuran (28.7), 10-epi γ-eudesmol (13.5), 4-epi-*cis*-dihydro-agarofuran (10.8)
*Piper cernuum* Vell.	R1741/IAC 7268	Pariquera-Açu (47°52′48.76″ S, 24°36′48.42″ W, 25 m)	1.84	dihydro-β-agarofuran (33.8), 10-epi-γ-eudesmol (12.2), α-pinene (11.8), camphene (8.7)
*Piper crassinervium* Kunth.	R1764/IAC 7513	Mococa (46°59′55.30″ S, 21°25′24.56″ W, 568 m)	0.53	β-pinene (11.6), α-pinene (11.5), germacrene D (9,2), *trans*-caryophyllene (7.8), guaiol (5.5), bicyclogermacrene (5.1)
*Piper gaudichaudianum* Kunth.	R1738/IAC 7265	Pariquera-Açu (47°52′48.76″ S, 24°36′48.42″ W, 25 m)	0.16	*trans*-nerolidol (17.5), α-pinene (12.2), caryophyllene oxide (8.5), *trans*-caryophyllene (8.2), β-pinene (7.0), *trans*-β-guaiene (6.9)
*Piper leptorum* Kunth.	Lp052903/IAC 7085	Monte Alegre do Sul (46°39′53.99″ S, 22°42′13.32″ W, 778 m)	0.60	seychellene (34.7), caryophyllene oxide (12.5)
*Piper rivinoides* Kunth.	L52007/IAC 47078	Ubatuba (45°7′16.03″ S, 23°25′16.36″ W, 30 m)	0.63	α-pinene (73.2), β-pinene (5.2)
*Piper solmsianum* C.DC.	R1633/IAC 46832	Ubatuba (45°7′8.79″ S, 23°24′32.47″ W, 40 m)	0.39	δ-3-carene (66.9), myrcene (26.1), α-pinene (22.7), α-selinene (5.5)
*Piper umbellatum* (L.)	R4169/IAC 46978	Campinas (47°4′4.69″ S, 22°51′54.60″ W, 667 m)	0.18	germacrene D (55.8), bicyclogermacrene (11.8), *trans*-caryophyllene (6.3)
*Piper xylosteoides* (Kunth.) Steud.	L52004/IAC 47075	Ubatuba (45°7′37.64″ S, 23°25′16.03″ W, 30 m)	1.04	spathulenol (12.3), germacrene B (10.6), β-copaen-4-α-ol (9.4), *trans*-nerolidol (8.2), *trans*-β-guaiene; (7.8)
Rutaceae				
*Esenbeckia febrifuga* (A.St.-Hil.) A.Juss. ex Mart	Lp06091205/IAC 44591	Campinas (47°4′3.49″ S, 22°51′47.19″ W, 672 m)	0.14	caryophyllene oxide (46.7)
*Helietta apiculata* Benth.	Lp6101823/IAC 47989	Adamantina (51°9′12.19″ S, 21°39′41.90″ W, 365 m)	0.16	limonene (42.3)
*Metrodorea nigra* A.St.-Hil.	Lp06091204/IAC 46826	Campinas (47°4′0.52″ S, 22°51′52.24″ W, 650 m)	0.05	spathulenol (23.6), bicyclogermacrene (16.6), germacrene D (15.3)
*Zanthoxylum petiolare* A.St.-Hil. & Tul.	Lp6101710/IAC 47962	Votuporanga (50°3′29.80″ S, 20°27′27.40″ W, 479 m)	0.18	β-phellandrene (40.7), germacrene D (22.0)
Salicaceae				
*Casearia sylvestris* Sw.	R1643/IAC 46842	Ubatuba (45°7′26.44″ S, 23°24′37.87″ W, 50 m)	0.16	*trans*-β-guaiene (12.2), 1,10-di-epi-cubenol (12.1)
Sapindaceae				
*Cupania vernalis* Cambess.	L4160/IAC 46969	Campinas (47°4′1.63″ S, 22°51′47.24″ W, 670 m)	0.20	bicyclogermacrene (35.9), germacrene D (21.4), *trans*-caryophyllene (16.1)
Verbenaceae				
*Aloysia virgata* (Ruiz & Pav.) Juss.	C004/IAC 4614	Jundiai (46°55′40.45″ S, 23°6′42.48″ W, 770 m)	0.22	γ-muurolene (32.7), *trans*-β-guaiene (24.6)

**Table 2:** Minimum inhibitory concentrations (MIC) of selected EOs. Growth inhibition is presented in μg mL^−1^ in comparison to normalized positive and negative controls plus/minus standard errors.

		Microorganism							
Botanical Family		Bacteria						Filamentous Fungus	Yeast
Plant Species (Location)	Sample Code	*C. xerosis*	*E. coli*	*P. acnes*	*P. aeruginosa*	*S. aureus*	*S. epidermidis*	*A. niger*	*C. albicans*
Annonaceae									
*Xylopia brasiliensis* (Pariquera-Açu)	R1739	50 ± 2.2	50 ± 2.5	25 ± 1.3	25 ± 1.1	50 ± 2.5	50 ± 2.4	25 ± 1.2	25 ± 1.3
*Xylopia aromatica* (Votuporanga)	Lp6101712	6.2 ± 0.1	25 ± 1.2	12.5 ± 0.6	25 ± 1.2	25 ± 1.3	25 ± 1.2	25 ± 1.2	25 ± 1.2
Lauraceae									
*Nectandra megapotamica* (Ribeirão Preto)	R1774	6.2 ± 0.1	12.5 ± 0.6	25 ± 1.2	25 ± 1.1	25 ± 1.2	25 ± 1.2	25 ± 1.1	25 ± 1.2
Myrtaceae									
*Eugenia neoverrucosa* (Campinas)	R1626	6.2 ± 0.1	25 ± 1.2	25 ±1.2	25 ± 1.2	25 ± 1.1	25 ± 1.2	25 ± 1.2	25 ± 1.2
*Eugenia prasina* (Ubatuba)	Lp51905	12.5 ± 0.6	25 ± 1.2	25 ± 1.2	12.5 ± 0.6	25 ± 1.1	25 ± 1.1	25 ± 1.2	25 ± 1.2
*Eugenia pyriformis* (Jundiai)	C009	50 ± 2.3	50 ± 2.2	25 ± 1.2	25 ± 1.2	50 ± 2.2	50 ± 2.3	25 ± 1.2	25 ± 1.2
*Myrcia splendens* (Jundiai)	C007	12.5 ± 0.6	25 ± 1.2	12.5 ± 0.6	25 ± 1.2	25 ± 1.2	25 ± 1.2	25 ± 1.2	25 ± 1.2
Rutaceae									
*Helietta apiculata* (Adamantina)	Lp6101823	12.5 ± 0.6	25 ± 1.2	12.5 ± 0.6	25 ± 1.2	25 ± 1.2	25 ± 1.2	25 ± 1.2	25 ± 1.2
Salicaceae									
*Casearia sylvestris* (Ubatuba)	R1643	12.5 ± 0.6	25 ± 1.2	12.5 ± 0.5	25 ± 1.2	25 ± 1.3	25 ± 1.2	25 ± 1.2	25 ± 1.2

## Data Availability

The metabolomics and metadata reported in this paper are available at Metabolomics Workbench (https://www.metabolomicsworkbench.org/data/index.php (accessed on 7 December 2022)), study identifier ST000606.

## Supplementary Materials

The following supporting information can be downloaded at: https://www.mdpi.com/article/10.3390/antibiotics11121844/s1, Figure S1: Biogeographically defined domain of the Atlantic rainforest in Brazil and plant collection sites in the state of São Paulo; Figure S2: Distribution of plants from botanical families in the Rainforest locations; Figure S3: Essential oil yield per botanical family, location, season, and plant growth habit; Figure S4: PCA of the chemical composition of the EOs; Figure S5: Performance of sPLS-DA of the EO chemical composition using botanical families as discriminant; Figure S6: Bayesian Information Criterion (BIC) and Gaussian Mixture Model (GMM) classification of the metabolic profile of EOs; Figure S7: Growth inhibition (%) of the EOs in agar diffusion assays; Table S1: Chemical composition and metabolite identification for the EOs from 63 plants from the Atlantic Rainforest; Table S2: Correlation analyses between the contents of the most abundant metabolites from the isolated EOs and growth inhibition; Table S3: Correlation analyses between cell component loss and growth inhibition; Table S4: Prediction of pharmacokinetic properties of 27 major components of the EOs. Table S5. Agglomerate macromolecular target prediction for the major metabolites of the EOs from the Annonaceae, Lauraceae, Myrtacea, Rutaceae, and Salicaceae botanical families. Table S6. Botanical classification, biome of occurrence, aroma description, antimicrobial action, and reported EO toxicity of the plants sampled in Atlantic Rainforest locations.
