# Supplementary material for: Essential Oils of Aromatic Plant Species from the Atlantic Rainforest Exhibit Extensive Chemical Diversity and Antimicrobial Activity
Source: Antibiotics (Basel). 2022 Dec 19;11(12):1844. doi: 10.3390/antibiotics11121844 (PMC9774909; doi:10.3390/antibiotics11121844)
Supplement: Supplementary file 1 [file antibiotics-11-01844-s001.zip › Supplementary Material.pdf]

**Supplementary Material**

**Supplementary Tables**

**Table S1.** Chemical composition and metabolite identification for the EOs from 63 plants from the Atlantic Rainforest. Complete data and corresponding metadata are available Metabolomics Workbench (https://www.metabolomicsworkbench.org/data/index.php), study identifier ST000606.

**Table S2.** Correlation analyses between the contents of the most abundant metabolites from the isolated EOs and growth inhibition against *E. coli*, *S. epidermidis*, *S. aureus* and *C. xerosis*. Pearson *r* and correlation *p*-value are presented.

**Table S3**. Correlation analyses between cell component loss and growth inhibition against *E. coli*, *S. epidermidis*, *S. aureus* and *C. xerosis*. Leakage of cellular components is shown for nucleic acid (na) and protein (prot). Growth inhibition is represented by gi. Bacteria species are represented by abbreviations: *E. coli*, *E.* coli; *S. epidermidis*, *S. epi*; *S. aureus*, Sau; *Cxer*, *C. xerosis*. Pearson *r* and correlation *p*-value are presented.

**Table S4**. Prediction of pharmacokinetic properties of 27 major components of the EOs isolated from aromatic species from the Atlantic rainforest.

**Table S5**. Agglomerate macromolecular target prediction for the major metabolites of the EOs from the Annonaceae, Lauraceae, Myrtacea, Rutaceae, and Salicaceae botanical families.

**Table S6.** Botanical classification, biome of occurrence, aroma description, antimicrobial action, and reported EO toxicity of the plants sampled in Atlantic Rainforest locations. Antimicrobial action and non-target toxicity information were retrieved from database and search engine queries from 2010 to 2022. Investigated databases were Agris (http://agris.fao.org), AGRICOLA (https://agricola.nal.usda.gov), Scopus (https://www.scopus.com), PubMed (http://pubmed.ncbi.nlm.nih.gov), and the core collection of Web of

Science (https://apps.webofknowledge.com), and search engines were Google Scholar (https://scholar.google.no/) and JSTOR (jstor.org). No report retrieved is shown as information not available (na).

**Supplementary Figure Captions**

**Fig. S1** Biogeographically defined domain of the Atlantic rainforest in Brazil (**A**) and plant collection sites in the state of São Paulo (**B**). Satellite images obtained from Google Earth.

**Fig. S2** Distribution of plants (number of plants) from 15 botanical families in the nine Rainforest locations investigated. Locations are identified on the top part of the graphs, and botanical families are represented by colors.

**Fig. S3** Essential oil yield ($g.g^{-1}$ per-mille) discriminated according to the botanical family (**A**), location (**B**), season (**C**) and plant growth habit (D). Locations are identified by the abbreviations: Ada, Adamantina; Cam, Campinas; Jun, Jundiaí; Moc, Mococa; MoA, Monte Alegre do Sul; Par, Pariquera-Açu; Rib, Ribeirão Preto; Uba, Ubatuba; Vot, Votuporanga.

**Fig. S4** Principal component analysis (PCA) of the chemical composition of the EOs from aromatic plants of the Atlantic Rainforest. (**A**) Scree and (**B**) variable factor plots.

**Fig. S5** Performance of sparse Partial Least Square Discriminant Analysis (sPLS-DA), using botanical family as discriminant with balanced data (**A**); error rates calculated by centroid, Mahalanobis and maximum distances (**B**), and Receiver Operator Characteristic (ROC) curve for the sPLS-DA component 3 (**C**). Distances used to calculate error rates are present on graphs in light orange bars.

**Fig. S6** Bayesian Information Criterion (BIC) and Gaussian Mixture Model (GMM) classification of the metabolic profile of EOs from Annonaceae (**A**), Asteraceae (**B**), Euphorbiaceae (**C**), Lauraceae (**D**), Myrtaceae (**E**), Piperaceae (**F**) and Rutaceae (**G**).

**Fig. S7** Percentage growth inhibition bacterial by the EO from native rainforest species from the Atlantic rainforest in the state of São Paulo against one Gram negative (E. coli) and three Gram-positive (S. epidermidis, S. aureus and C. xerosis) bacterial pathogens. Inhibition percentage was calculated based on the recommended concentration (500 μg mL−1) of wide spectrum commercial antibiotic cefotaxime. Sterile mineral oil was used as negative control.
