# Supplementary material for: Essential Oils of Aromatic Plant Species from the Atlantic Rainforest Exhibit Extensive Chemical Diversity and Antimicrobial Activity
Source: Antibiotics (Basel). 2022 Dec 19;11(12):1844. doi: 10.3390/antibiotics11121844 (PMC9774909; doi:10.3390/antibiotics11121844)
Supplement: Supplementary file 1 [file antibiotics-11-01844-s001.zip › Table S6.pdf]

Table S6. Botanical classification, biome of occurrence, aroma description, antimicrobial action, and reported EO toxicity of the plants sampled in Atlantic Rainforest locations. Antimicrobial action and non-target toxicity information were retrieved from database and search engine queries from 2010 to 2022. Investigated databases were Agris (http://agris.fao.org), AGRICOLA (https://agricola.nal.usda.gov), Scopus (https://www.scopus.com), PubMed (http://pubmed.ncbi.nlm.nih.gov), and the core collection of Web of Science (https://apps.webofknowledge.com), and search engines were Google Scholar (https://scholar.google.no/) and JSTOR (jstor.org). No report retrieved is shown as information not available (na).

**Botanical Family**	**Genera**	**Biome/Aroma descriptor**	**Antimicrobial action**	**Toxicity (non-target)**	**References**
Anacardiaceae	*Schinus*	Cerrado, Atlantic rainforest/ waxy, herbal, woody green	antibacterial, antifungal, insecticidal, antiparasitic, disinfectant, oral hygiene	sesquiterpenes, *in vitro* [1]; α-phellandrene, *in vitro* [2]	[3, 4, 5, 6]
Annonaceae	*Annona*, *Guatteria*, *Xylopia*	Cerrado/ sweet, pungent, bitter, oily	antibacterial, antifungal, antiparasitic	spathulenol, caryophyllene oxide, α-pinene and byciclogermacrene [7]; germacrene D, bicyclogermacrene, (E)-caryophyllene and germacrene B [8]; α-pinene, limonene,*in vivo*, no genotoxicity detected [9]	[7, 8, 10]
Araceae	*Monstera*	Atlantic and Amazon ainforest/ fruity, sweet,	antibacterial, antifungal, insecticidal, antiparasitic,	na	[11, 12]

		alcoholic, spicy	disinfectant		
Araliaceae	*Dendropanax*	Cerrado, Atlantic rainforest/ herbal, alcoholic, woody green	antibacterial, antifungal, insecticidal, antiparasitic, antiviral (Asia)	na	[13, 14]
Asteraceae	*Baccharis*, *Cyrtocymura*	Cerrado/ sweet, resinous, woody	antibacterial, antifungal, antiviral, skin hygiene	carquejyl acetate, *in vivo* study, no toxicity detected [15]	[16, 17, 18]
Euphorbiaceae	*Croton*	Cerrado, Atlantic rainforest/ pine needle-like, herbal, oily, woody green	antibacterial, antifungal, insecticidal, antiparasitic	eugenol, methyl-eugenol, no toxicity detected [19];anethole and estragole, *in vivo* study, tubule-interstitial injury [20]	[21, 22]
Lauraceae	*Aiouea*, *Aniba*, *Endlicheria*, *Nectandra*, *Ocotea*	Cerrado, Atlantic and Amazon rainforest/ cinnamon-like, woody, sweet, oily, fruit	antibacterial, antifungal, antiviral, skin and oral hygiene	1-nitro-2-phenylethane, methyleugenol, eugenol, safrol,*in vivo*, no toxicity detected [23]	[24, 25, 26, 27]
Meliaceae	*Trichilia*	Cerrado, Atlantic and Amazon rainforest/ sweet, fruity, oily, citrus-like	antibacterial, antifungal, insecticidal, antiparasitic	na	[28, 29]
Myristicaceae	*Virola*	Atlantic and Amazon rainforest/ flowery, sweet,	antibacterial, antifungal, antiparasitic	na	[30, 31, 32]

		citrus-like			
Myrtaceae	*Calyptranthes, Campomanesia, Eugenia, Marlierea, Myrcia*	Cerrado, Atlantic rainforest/ fruity, sweet	antibacterial, antifungal,antiparasitic	(Z)-α-trans-bergamotene, α-sinensal, (Z)-α-bisabolene, *in vitro*, high toxicity [33];β-caryophyllene, δ-cadinene, α-pinene, β-pinene, moderate toxicity, eliminated in nanoemulsion [34]	[35, 36, 37, 38]
Piperaceae	*Piper*	Cerrado, Atlantic and Amazon rainforest/ pepper-like, herbal, green	antibacterial, antifungal, insecticidal, antiparasitic	β-caryophyllene, α-humulene, and germacrene D, no toxicity detected [39];*in vivo*, no toxicity detected [40]	[41, 42, 43, 44]
Rutaceae	*Esenbeckia, Helietta, Metrodorea, Zanthoxylum*	Cerrado, Atlantic rainforest/ pine needle-like, citrus-like, spicy, woody	antibacterial, antifungal, insecticidal, antiparasitic, disinfectant, skin and oral hygiene	linalyl acetate, limonene, and α-terpineol, no detected toxicity as nanoemulsion [45]; limonene, β-pinene, *in vivo*, no toxicity detected [46]	[47, 48, 49, 50, 51]
Salicaceae	*Casearia*	Cerrado, Atlantic and Amazon	antibacterial, antifungal,	β-caryophyllene, β-humulene,	[53, 54]

		rainforest/ pine needle-like, spicy, herbal, woody green	antiparasitic	selective cytotoxicity to tumoral cells [52]	
Sapindaceae	*Cupania*	Atlantic and Amazon rainforest/ nut-like, woody, oily, spicy	antibacterial, antifungal	na	[11, 55, 56]
Verbenaceae	*Aloysia*	Atlantic and Amazon rainforest/ citrus-like, fruity, flower, herbal, pine needle-like	antibacterial, antifungal, antiparasitic	(E)-caryophyllene, bicyclogermacrene, germacrene D, terpinolene, and sabinene, *in vitro*, toxicity to fibroblasts [57];linalool, geraniol, neral, and limonene, *in vivo*, no toxicity detected [58]	[59, 60, 61]

References

1. Mattar, V.T.;Borioni, J.L.; Hollmann, A.; Rodriguez, S.A. Insecticidal activity of the essential oil of *Schinusareira* against *Rhipibruchuspicturatus* (F.) (Coleoptera: Bruchinae), and its inhibitory effects on acetylcholinesterase. *PesticBiochem Physiol.***2022**, 185, 105134. doi: 10.1016/j.pestbp.2022.105134.
2. de Groot, A.C.; Schmidt, E. Tea tree oil: contact allergy and chemical composition. *Contact Dermatitis***2016**,75:129-143. doi: 10.1111/cod.12591.

3. Moura-Costa, G.F.; Nocchi, S.R.;Ceole, L.F.; de Mello, J.C.P.; Nakamura, C.V.; Dias Filho, P.B.; Temponi, L.G.; Ueda-Nakamura, T. Antimicrobial activity of plants used as medicinals on an indigenous reserve in Rio das Cobras, Paraná, Brazil.*J. Ethnopharmacol*. **2012**, 143, 631-638. doi:https://doi.org/10.1016/j.jep.2012.07.016.
4. Gehrke, I. T.; Neto, A. T.; Pedroso, M.;Mostardeiro, C. P.;da Cruz, I. B.; Silva, U. F.;Ilha, V.;Dalcol, I. I.; Morel, A. F. Antimicrobial activity of *Schinuslentiscifolius* (Anacardiaceae). *J Ethnopharmacol*. **2013**, 148, 486–491. https://doi.org/10.1016/j.jep.2013.04.043.
5. Uliana, M.P.; Fronza, M.; da Silva, A.G.; Vargas, T.S.; de Andrade, T.U.; Scherer, R. Composition and biological activity of Brazilian rose pepper (*Schinus terebinthifolius* Raddi) leaves. *Ind. Crops Prod.***2016**, 83, 235-240. doi: https://doi.org/10.1016/j.indcrop.2015.11.077.
6. de Souza Feitosa Lima, I.M.; Zagmignan, A.; Santos, D.M.; Maia, H.S.; dos Santos Silva, L.; da Silva Cutrim, B.; Vieira, S.L.; BezerraFilho, C.M.; de Sousa, E.M.; Napoleão, T.H.; Krogfelt, K.A.; Løbner-Olesen, A.;Paiva, P.M.G.; Nascimento da Silva, L.C. *Schinus terebinthifolia* leaf lectin (SteLL) has anti-infective action and modulates the response of *Staphylococcus aureus*-infected macrophages. *Sci Rep*.**2019**, 9, 18159. doi: 10.1038/s41598-019-54616-x.
7. Cascaes, M.M.; De Moraes, Â.A.B.; Cruz, J.N.; Franco, C.J.P.E.; Silva, R.C.; Nascimento, L.D.D.; Ferreira, O.O.; Anjos, T.O.D.; de Oliveira, M.S.;Guilhon, G.M.S.P.; Andrade, E.H.A. Phytochemical profile, antioxidant potential and toxicity evaluation of the essential oils from *Duguetia* and *Xylopia* species (Annonaceae) from the Brazilian Amazon. *Antioxidants* (Basel). **2022**, 11, 1709. doi: 10.3390/antiox11091709.
8. Pereira, T.S., Machado Esquissato, G.N., Costa, E.V., Nogueira, P.C.L., Castro-Prado, M.A.A. Mutagenic and cytostatic activities of the *Xylopialaevigata* essential oil in human lymphocytes. *Nat Prod Res.***2021**, 35, 1682-1685. doi: 10.1080/14786419.2019.1624956.
9. Moura, A.P.; Beltrão, D.M.; Pita, J.C.; Xavier, A.L.; Brito, M.T.; Sousa, T.K.; Batista, L.M.; Carvalho, J.E.; Ruiz, A.L.; Della Torre, A.; Duarte, M.C.; Tavares, J.F.; da Silva, M.S.; Sobral, M.V. Essential oil from fruit of *Xylopialangsdorffiana*: antitumour activity and toxicity. *Pharm Biol*. **2016**, 54, 3093-3102. doi:10.1080/13880209.2016.1211154.

10. Nascimento, M.N.G.; Junqueira, J.G.M.; Terezan, A.P.; Severino, R.P.; Silva, T.S.; Martins, C.H.G.; Severino, V.G.P. Chemical composition and antimicrobial activity of essential oils from *Xylopiaaromatica* (Annonaceae) flowers and leaves. *Rev. Virtual Quim*.**2018**, 10, 1578-1590. doi: 10.21577/1984-6835.20180107

11. Ricardo, L.M., de Paula-Souza, J., Andrade, A., Brandão, M.G.L. Plants from the Brazilian traditional medicine: species from the books of the Polish physician Piotr Czerniewicz (Pedro Luiz NapoleãoChernoviz, 1812–1881). *Rev. Bras. Farmacog*. **2017**, 27, 388-400. doi: https://doi.org/10.1016/j.bjp.2017.01.002.

12. Meneguelli, A.Z.; Camargo, E.E.S.; Buccini, D.F.; Roriz, B.C.; Cerqueira, G.R.; Moreno, S.E. Ethnopharmacological and botanical evaluation of medicinal plants used by Brazilian Amazon Indian community. *Interações* (Campo Grande)**2020**, 21, 3. doi: https://doi.org/10.20435/inter.v21i3.2926

13. Cruz-Galvez, A.M.; Gómez-Aldapa, C.A.; Villagómez-Ibarra, J.R.; Chavarría-Hernández, N.; Rodríguez-Baños, J.; Rangel-Vargas, E.; Castro-Rosas, J. Antibacterial effect against foodborne bacteria of plants used in traditional medicine in central Mexico: studies in vitro and in raw beef. *Food Control***2013**, 32, 289-295. doi: https://doi.org/10.1016/j.foodcont.2012.12.018.

14. Balakrishnan, R.; Cho, D.-Y.; Su-Kim, I.; Choi, D.-K. *Dendropanaxmorbiferus* and other species from the genus *Dendropanax*: therapeutic potential of its traditional uses, phytochemistry, and pharmacology. *Antioxidants***2020**, 9, 962. doi: https://doi.org/10.3390/antiox9100962

15. Bueno, G.; Chavez Rico, S.L.;Périco, L.L.;Ohara, R.; Rodrigues, V.P.;Emílio-Silva, M.T.;Assunção, R.; Machado da Rocha, L.R.;Nunes, D.S.;Besten, M.A.;Heiden, G.; Lima Camargo, A.C.;Justulin, L.A.;Hiruma-Lima, C.A. The essential oil from *Baccharistrimera* (Less.) DC improves gastric ulcer healing in rats through modulation of VEGF and MMP-2 activity. *J Ethnopharmacol*. **2021**, 271:113832. doi: 10.1016/j.jep.2021.113832.

16. Cazella, L.N.; Glamoclija, J.;Soković, M.; Gonçalves, J.E.; Linde, G.A.; Colauto, N.B.; Gazim, Z.C. Antimicrobial activity of essential oil of *Baccharisdracunculifolia* DC (Asteraceae) aerial parts at flowering period. *Front. Plant Sci*. **2019**, 10, 27. doi: 10.3389/fpls.2019.00027

17. Zuccolotto, T.; Bressan, J.; Lourenço, A.V.F.; Bruginski, E.;Veiga, A.; Marinho, J.V.N.; Raeski, P.A.; Heiden, G.; Salvador, M.J.; Murakami, F.S.; Budel, J.M.; Campos, F.R. Chemical, antioxidant, and antimicrobial evaluation of essential oils and an anatomical study of the aerial parts from *Baccharis* species (Asteraceae). *Chem. Biodiv*. **2019**, 16, e1800547. doi: https://doi.org/10.1002/cbdv.201800547

18. Camargo, J.G.S.D. Desreplicação dos extratosativos de *Cyrtocymurascorpioides* (Asteraceae) contra *Candida* spp. e *Trichomonas vaginalis*. **2020**. MSc. dissertation, Instituto de Biociências, Letras e CiênciasExatas (IBILCE), UNESP, São José do Rio Preto, SP, Brazil. available at: <http://hdl.handle.net/11449/194314

19. Santos, N.C.; Silva, J.E.D.; Santos, A.C.C.;Dantas, J.O.; Tavares, S.R.S.A.; Andrade, V.S.; Oliveira, S.D.D.S.; Blank, A.F.;Araújo, A.P.A.;Bacci, L. Bioactivity of essential oils from *Croton grewioides* and its major compounds: toxicity to soybean looper*Chrysodeixisincludens* and selectivity to the predatory stink bug *Podisusnigrispinus*. *Environ SciPollut Res Int*. **2022**. doi: 10.1007/s11356-022-23414-w.

20. Silva, K.F.;Peruchetti, D.B.;Sirtoli, G.M.;Takiya, C.M.;Pinheiro, A.A.S.; Leal-Cardoso, J.H.; Caruso-Neves, C. High doses of essential oil of *Croton zehntneri* induces renal tubular damage. *Plants* (Basel) **2021**, 10, 1400. doi: 10.3390/plants10071400.

21. Almeida-Pereira, C.S.; Nogueira, P.C.L.; Barbosa, A.A.T.; Nizio, D.A.C.; Arrigoni-Blank, M.F.; Sampaio, T.S.; Alves, R.P.; de Araujo-Couto, H.G.S.; Feitosa-Alcantara, R.B.; de Melo, J.O.; Blank, A.F. Chemical composition and antimicrobial activity of essential oils of a *Croton tetradenius*Baill. germplasm. *J. Essent. Oil Res.***2019**, 31, 379-389. doi: 10.1080/10412905.2019.1607785

22. Rocha, A.R.F.S.; Sousa, H.G.; do Vale Júnior, E.P.; de Lima, F.L.; Costa, A.S.G.; de Araújo, A.R.; Leite, J.R.S.A.; Martins, F.A.; Oliveira, M.B.P.P.; Plácido, A.; Filho, F.S.S.; Lago, E.C. Extracts and fractions of *Croton* L. (Euphorbiaceae) species with antimicrobial activity and antioxidant potential. *LWT***2021**, 139, 110521. doi: https://doi.org/10.1016/j.lwt.2020.110521.

23. Silva, N.N.; Silva, J.R.; Alves, C.N.; Andrade, E.H.; da Silva, J.K.; Maia, J.G. Acetylcholinesterase inhibitory activity and molecular docking study of 1-nitro-2-phenylethane, the main constituent of *Anibacanelilla* essential oil. *Chem Biol Drug Des*. **2014**, 84, 192-8. doi: 10.1111/cbdd.12304.

24. da Silva, J.K.R.; da Trindade, R.C.S.; Maia, J.G.S.; Setzer, W.N. Chemical composition, antioxidant, and antimicrobial activities of essential oils of *Endlicheriaarenosa* (Lauraceae) from the Amazon. *Nat. Prod. Commun*. **2016**, 11, 695–698. doi: 10.1177/1934578X1601100537

25. de Moura, V.M.; Guimarães, N.C.; Batista, L.T.; Freitas-de-Sousa, L.A.; Martins, J.S.; de Souza, M.C.S.; de Almeida, P.D.O; Monteiro, W.M.; de Oliveira, R.B.; dos-Santos, M.C.; Mourão, R.H.V. Assessment of the anti-snakebite properties of extracts of *Anibafragrans*Ducke (Lauraceae) used in folk medicine as complementary treatment in cases of envenomation by *Bothropsatrox*, *J. Ethnopharmacol*. **2018**, 213, 350-358. doi: https://doi.org/10.1016/j.jep.2017.11.027.

26. da Silva, Y.C.; Silva, E.M.S.; Fernandes, N.S.; Lopes, N.L.;Orlandi, P.P.; Nakamura, C.V.; Costa, E.V.; da VeigaJúnior, V.F. Antimicrobial substances from Amazonian *Aniba* (Lauraceae) species. *Nat. Prod. Res*. **2021**, 35, 849-852. doi: 10.1080/14786419.2019.1603225

27. Xavier, J.K.A.M.; Alves, N.S.F.; Setzer, W.N.; da Silva, J.K.R. Chemical diversity and biological activities of essential oils from *Licaria*, *Nectrandra* and *Ocotea* species (Lauraceae) with occurrence in Brazilian biomes. *Biomolecules***2020**, 10, 869. doi: https://doi.org/10.3390/biom10060869

28. Longhini, R.; Lonni, A.A.; Sereia, A.L.; Krzyzaniak, L.M.; Lopes, G.C.; Mello, J.C.P.D. *Trichiliacatigua*: therapeutic and cosmetic values. *Rev. Bras. Farmacog*. **2017**, 27, 254-271. doi: https://doi.org/10.1016/j.bjp.2016.10.005

29. da Silva, L.L.; de Almeida R.; Silva, F.T.E.; Verícimo, M.A. Review on the therapeutic activities of the genus *Trichilia*. *Res. Soc. Dev*. **2021**, 10, e29610514916. doi: 10.33448/rsd-v10i5.14916.

30. Cordeiro, R.M.; de S. e Silva, A.P.; Pinto, R.H.; da Costa, W.A.; da Silva, S.H.; de Souza Pinheiro, W.B.; Arruda, M.S.P.; Carvalho Junior, R.N. Supercritical CO_2 extraction of ucuúba (*Virolasurinamensis*) seed oil: global yield, kinetic data, fatty acid profile, and antimicrobial activities. *Chem. Eng. Commun*. **2019**, 206, 86-97. doi: https://doi.org/10.1080/00986445.2018.1474741

31. González-Rodríguez, M.; Ruiz-Fernández, C.; Francisco, V.; AitEldjoudi, D.;FarragAbd-El-Hafez ,Y.R.; Cordero-Barreal, A.; Pino, J.; Lago, F.; Campos-Toimil, M.; Rocha Carvalho, G.; Melo Costa Pereira, T.; Gualillo, O. Pharmacological extracts and molecules from *Virola*species: Traditional uses, phytochemistry, and biological activity. *Molecules***2021**, 26, 792. doi: https://doi.org/10.3390/molecules26040792

32. Ribeiro, C.L.; Silva, R.M.; Fernandes, R.D.M.N.; Araújo, M.R.; Soares, I.M.; da Silva, J.F.M.; do Nascimento, G.N.L.; Pimenta, R.S.; Scapin, E. Chemical assessment, antioxidant and antimicrobial of leafs extracts of *Virolasebifera*, an Amazonian medicinal plant. *Res. Soc. Dev*. **2021**, 10, e578101523068-e578101523068. doi: http://dx.doi.org/10.33448/rsd-v10i15.23068

33. de Moraes, Â.A.B.; Ferreira, O.O.; da Costa, L.S.; Almeida, L.Q.; Varela, E.L.P.;Cascaes, M.M.; de Jesus Pereira Franco, C.;Percário, S.; Nascimento, L.D.D.; de Oliveira, M.S.; Andrade, E.H.A. Phytochemical profile, preliminary toxicity, and antioxidant capacity of the essential oils of *Myrciaria floribunda* (H. West ex Willd.) O. Berg. and *Myrcia sylvatica* (G. Mey) DC. (Myrtaceae). *Antioxidants* (Basel). **2022**, 11, 2076. doi: 10.3390/antiox11102076.

34. Magalhães, B.Q.; Machado, F.P.;Sanches, P.S.; Lima, B.;Falcão, D.Q.; von Ranke, N.; Bello, M.L.; Rodrigues, C.R; Santos, M.G.; Rocha, L.;Faria, R.X. *Eugenia sulcata* (Myrtaceae) nanoemulsion enhances the inhibitory activity of the essential oil on P2X7R and inflammatory response *in vivo*. *Pharmaceutics***2022**, 14, 911. doi: 10.3390/pharmaceutics14050911.

35. de Araújo, F.F.; Neri-Numa, I.A.; Farias, D.P.; Castro da Cunha, G.R.M.; Pastore, G.M. Wild Brazilian species of *Eugenia* genera (Myrtaceae) as an innovation hotspot for food and pharmacological purposes. *Food Res. Intl*. **2019**, 121, 57-72. doi: https://doi.org/10.1016/j.foodres.2019.03.018.

36. Macedo, J.G.F.; Rangel, J.M.L.; de Oliveira Santos, M.; Camilo, C.J.; da Costa, J.G.M.; de Almeida Souza, M.M. Therapeutic indications, chemical composition and biological activity of native Brazilian species from *Psidium genus* (Myrtaceae): A review. *J. Ethnopharmacol*. **2021**, 278, 114248. doi: https://doi.org/10.1016/j.jep.2021.114248

37. Vechi, G.; Tenfen, A.; Capusiri, E.S.;Gimenez, A.; Cechinel-Filho, V. Antiparasitic activity of two Brazilian plants: *Eugenia mattosii* and *Marliereaeugeniopsoides*. *Nat. Prod. Res*. **2021**, 35, 4876-4880. doi: https://doi.org/10.1080/14786419.2020.1739676

38. Maiolini, T.C.S.; Rosa, Miranda, D.W.O.; Costa-Silva, T.A.; Tempone, A.G.; Pires Bueno, P.C.; Ferreira Dias, D.; de Paula, D.A.C.; Sartorelli, P.;Lago, J.H.G.; Gomes Soares, M. Essential oils from different Myrtaceae species from Brazilian Atlantic Forest Biome – chemical dereplication and evaluation of antitrypanosomal activity. *Chem. Biodiv*. **2022**, 19, e202200198. doi: https://doi.org/10.1002/cbdv.202200198

39. de Oliveira, A.C.;Simões, R.C.; Lima, C.A.P.; da Silva, F.M.A.;Nunomura, S.M.; Roque, R.A.;Tadei, W.P.;Nunomura, R.C.S. Essential oil of *Piper purusanum* C.DC (Piperaceae) and its main sesquiterpenes: biodefensives against malaria and dengue vectors, without lethal effect on non-target aquatic fauna. *Environ SciPollut Res Int*. **2022**, 29, 47242-47253. doi: 10.1007/s11356-022-19196-w.

40. Zhang, C.; Zhao, J.; Famous, E.; Pan, S.; Peng, X.; Tian, J. Antioxidant, hepatoprotective and antifungal activities of black pepper (*Piper nigrum* L.) essential oil. *Food Chem.***2021**, 346, 128845. doi: 10.1016/j.foodchem.2020.128845.

41. da Silva, J.K.; da Trindade, R.; Alves, N.S.; Figueiredo, P.L.; Maia, J.G.S.; Setzer, W.N. Essential oils from neotropical *Piper* species and their biological activities. *Intl. J. Mol. Sci*. **2017**, 18, 2571. doi: https://doi.org/10.3390/ijms18122571

42. de Pascoli, I.C.; dos Anjos, M.M.; da Silva, A.A.; Lorenzetti, F.B.; Cortez, D.A.G.; Mikcha, J.M.G.; Nakamura, T.U.; Nakamura, C.V.; de Abreu Filho, B.A. Piperaceae extracts for controlling *Alicyclobacillusacidoterrestris* growth in commercial orange juice. *Ind. Crops Prod*. **2018**, 116, 224-230. doi: https://doi.org/10.1016/j.indcrop.2018.02.073

43. Majolo, C.; Monteiro, P.C.; Nascimento, A.V.P.D.; Chaves, F.C.M.; Gama, P.E.; Bizzo, H.R.; Chagas, E.C. Essential oils from five Brazilian *Piper* species as antimicrobials against strains of *Aeromonashydrophila*. *J. Essent. Oil-Bear. Plants***2019**, 22, 746-761. doi:https://doi.org/10.1080/0972060X.2019.1645047

44. dos Santos, A.L.M.; Araújo, F.A.M.; da Silva Matisui, É.; da Costa, L.A.M.A.;Macedo, A.J.; de Lucena, J.M.V.M. Antimicrobial and antibiofilm properties of essential oils from *Piper marginatum* Jacq.. *Res. Soc. Dev*. **2021**, 10, e514101119967-e514101119967. doi: 10.33448/rsd-v10i11.19967.

45. NavaeiShoorvarzi, S.;Shahraki, F.;Shafaei, N.; Karimi, E.;Oskoueian, E. *Citrus aurantium* L. bloom essential oil nanoemulsion: Synthesis, characterization, cytotoxicity, and its potential health impacts on mice. *J Food Biochem*. **2020**, 44, e13181. doi: 10.1111/jfbc.13181.

46. Volpato, G.T.;Francia-Farje, L.A.;Damasceno, D.C.; Oliveira, R.V.;Hiruma-Lima, C.A.;Kempinas, W.G. Effect of essential oil from *Citrus aurantium* in maternal reproductive outcome and fetal anomaly frequency in rats. *An Acad Bras Cienc*. **2015**, 87, 407-15. doi: 10.1590/0001-3765201520140354.

47. Orlanda, J.F.;Nascimento, A.R.Chemical composition and antibacterial activity of *Rutagraveolens* L. (Rutaceae) volatile oils, from São Luís, Maranhão, Brazil. *South African J. Bot*. **2015**, 99, 103-106. doi: https://doi.org/10.1016/j.sajb.2015.03.198

48. Fernandes, T.S.; Copetti, D.; Do Carmo, G.; Neto, A.T.; Pedroso, M.; Silva, U.F.;Mostardeiro, M.A.; Burrow, R.E.;Dalcol, I.I.; Morel, A.F.Phytochemical analysis of bark from *Heliettaapiculata*Benth and antimicrobial activities. *Phytochemistry***2017**, 141, 131-139. doi: https://doi.org/10.1016/j.phytochem.2017.05.017

49. Silva, F.B.D.; Santos, N.O.D.;Pascon, R.C.;Vallim, M.A.;Figueiredo, C.R.; Martins, R.C.C.;Sartorelli, P.Chemical composition and *in vitro* cytotoxic and antimicrobial activities of the essential oil from leaves of *Zanthoxylummonogynum* St. Hill (Rutaceae). *Medicines***2017**, 4, 31. doi:https://doi.org/10.3390/medicines4020031

50. de Paula, R.C.; da Silva, S.M.;Faria, K.F.;Frézard, F.; de Souza Moreira, C.P.;Foubert, K.; Lopes, J.C.D.; Campana, P.R.V.; Rocha, M.P.; Silva, A.F.; Silva, C.G.In vitro antileishmanial activity of leaf and stem extracts of seven Brazilian plant species. *J. Ethnopharmacol*. **2019**, 232, 155-164. doi: https://doi.org/10.1016/j.jep.2018.12.026

51. Saraiva Filho, D.E.; Sousa, J.B.D.; Santos, H.S.D.; Fontenelle, R.O.D.S.Chemical compounds isolated from extracts and essential oils of the genus *Zanthoxylum* Linnaeus (Rutaceae) and its antimicrobial potential. *Hoehnea***2020**, 47, e882019. doi: https://doi.org/10.1590/2236-8906-88/2019.

52. Silva, S.L.D., Chaar, J.D.S., Figueiredo, P.D.M.S. and Yano, T., 2008. Cytotoxic evaluation of essential oil from CaseariasylvestrisSw on human cancer cells and erythrocytes. ActaAmazonica, 38, pp.107-112.

53. Pereira, F.G.;Marquete, R.; Cruz, L.O.;Caldeira-de-Arujo, A.; Mansur, E.; de Lima Moreira, D.DNA damages promoted by the essential oil from leaves of *Casearia sylvestris* Sw.(Salicaceae). *J. Med. Plant Res*. **2016**, 10, 818-822. doi: https://doi.org/10.5897/JMPR2016.6235

54. Ribeiro, S.M.;Fratucelli, É.; Bueno, P.C.; de Castro, M.K.V.; Francisco, A.A.;Cavalheiro, A.J.; Klein, M.I.Antimicrobial and antibiofilm activities of *Casearia sylvestris* extracts from distinct Brazilian biomes against *Streptococcus mutans* and *Candida albicans*. *BMC Complement. Alt. Med*. **2019**, 19, 1-16. doi: https://doi.org/10.1186/s12906-019-2717-z.

55. de O. Ribeiro, I.C.; Mariano, E.G.A.;Careli, R.T.;Morais-Costa, F.; de Sant'Anna, F.M.; Pinto, M.S.; de Souza, M.R.; Duarte, E.R.Plants of the Cerrado with antimicrobial effects against *Staphylococcus* spp. and *Escherichia coli* from cattle. *BMC Vet. Res*. **2018**, 14, 1-10. doi: https://doi.org/10.1186/s12917-018-1351-1

56. da Cruz, J.E.R.; Saldanha, H.C.; Freitas, G.R.O.;Morais, E.R.A review of medicinal plants used in the Brazilian Cerrado for the treatment of fungal and bacterial infections. *J. Herb. Med*. **2022**, 31, p.100523. doi: https://doi.org/10.1016/j.hermed.2021.100523.

57. Barros, L.M.; Duarte, A.E.;Morais-Braga, M.F.;Waczuk, E.P.; Vega, C.;Leite, N.F.; de Menezes, I.R.; Coutinho, H.D.; Rocha, J.B.;Kamdem, J.P. Chemical Characterization and trypanocidal, leishmanicidal and cytotoxicity potential of *Lantana camara* L. (Verbenaceae) essential oil. *Molecules*. **2016**, 21(2):209. doi: 10.3390/molecules21020209.

58. Postay, L.F.; Cabral, D.S.;Heringer, O.A.; Vieira, L.V.; de Moraes, L.R.; Freitas, G.; Gomes, L.C. The effectiveness of surfactants applied with essential oil of *Lippia alba* in the anesthesia of Nile tilapia (*Oreochromisniloticus*) and their toxicity assessment for fish and mammals. *Environ SciPollut Res Int*. **2021**, 28, 10224-10233. doi: 10.1007/s11356-020-11483-8.

59. de Souza, R.C.; Da Costa, M.M.;Baldisserotto, B.;Heinzmann, B.M.; Schmidt, D.; Caron, B.O.;Copatti, C.E.Antimicrobial and synergistic activity ofessential oils of *Aloysiatriphylla* and *Lippia alba* against *Aeromonas* spp.. *Microb. Pathog*. **2017**, 113, 29-33. https://doi.org/10.1016/j.micpath.2017.10.013.

60. Pérez Zamora, C.M.; Torres, C.A.;Nuñez, M.B.Antimicrobial activity and chemical composition of essential oils from Verbenaceae species growing in South America. *Molecules***2018**, 23, 544. doi: https://doi.org/10.3390/molecules23030544

61. Nader, T.T.; Leonel, A.H.; Henrique, C.Y.;Contini, S.H.T.;Crevelin, E.J.; de Castro França, S.; Berton, B.W.; Pereira, A.M.S.Dynamized*Aloysiapolystachya* (Griseb.) essential oil: a promising antimicrobial product. *Homeopath.***2022**.doi: 10.1055/s-0042-1748320
