# Supplementary figures and images for: Essential Oils of Aromatic Plant Species from the Atlantic Rainforest Exhibit Extensive Chemical Diversity and Antimicrobial Activity
Source: Antibiotics (Basel). 2022 Dec 19;11(12):1844. doi: 10.3390/antibiotics11121844 (PMC9774909; doi:10.3390/antibiotics11121844)

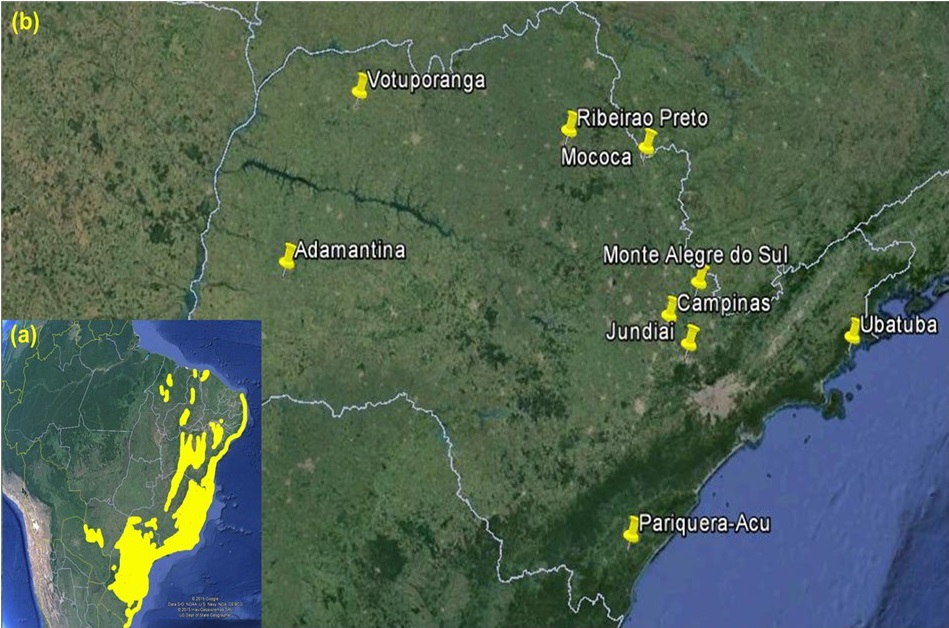

Supplement: Supplementary file 1 [file antibiotics-11-01844-s001.zip › FigureS1.jpg]

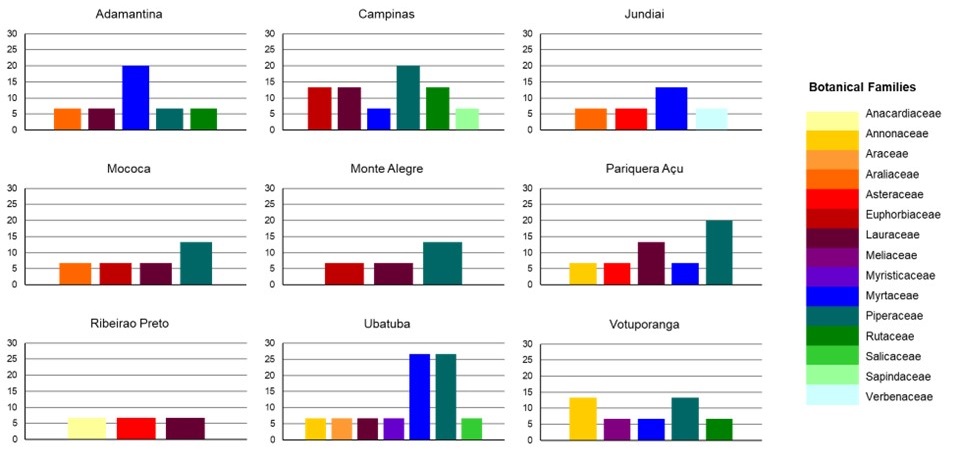

Supplement: Supplementary file 1 [file antibiotics-11-01844-s001.zip › FigureS2.jpg]

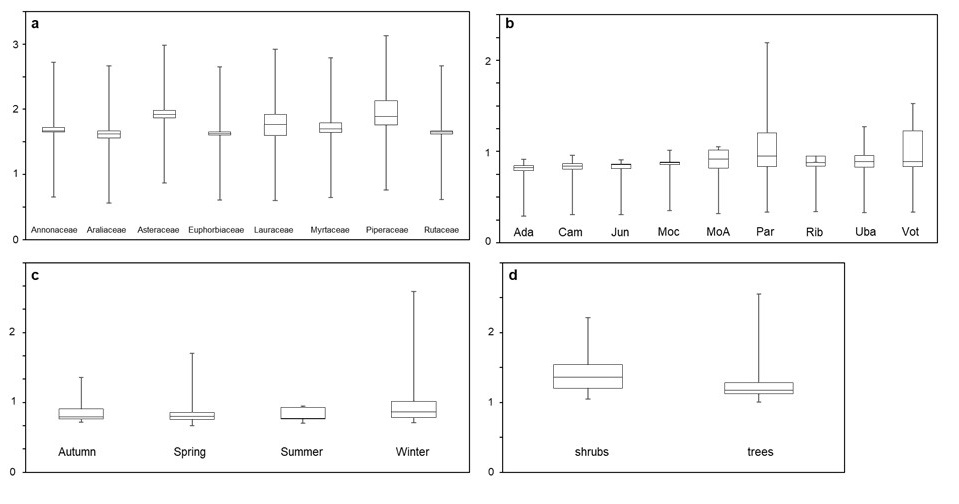

Supplement: Supplementary file 1 [file antibiotics-11-01844-s001.zip › FigureS3.jpg]

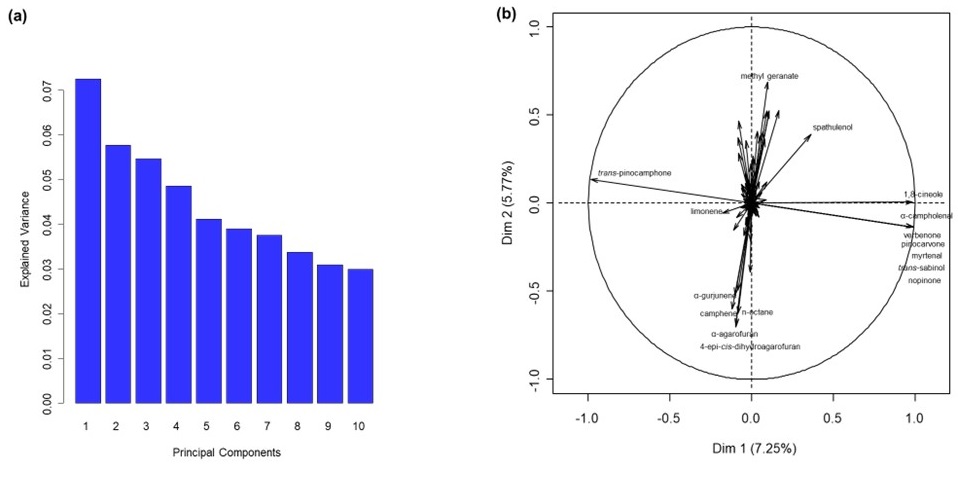

Supplement: Supplementary file 1 [file antibiotics-11-01844-s001.zip › FigureS4.jpg]

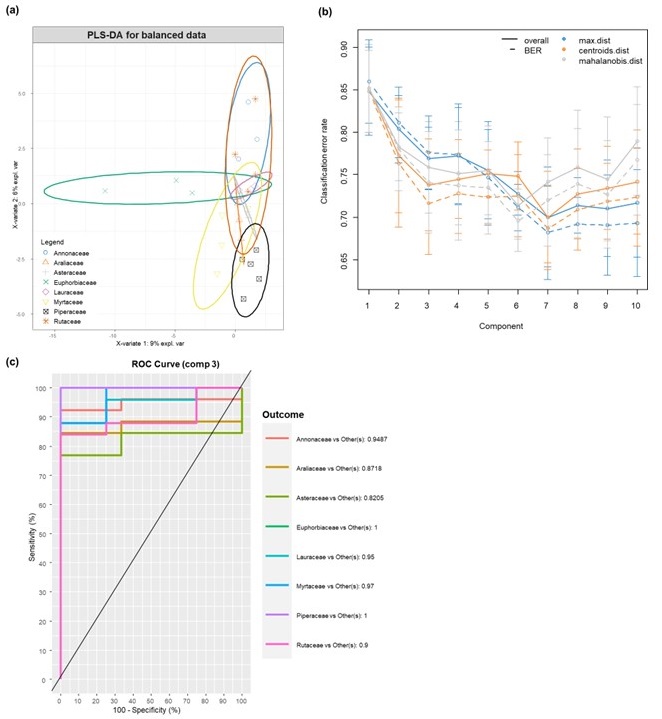

Supplement: Supplementary file 1 [file antibiotics-11-01844-s001.zip › FigureS5.jpg]

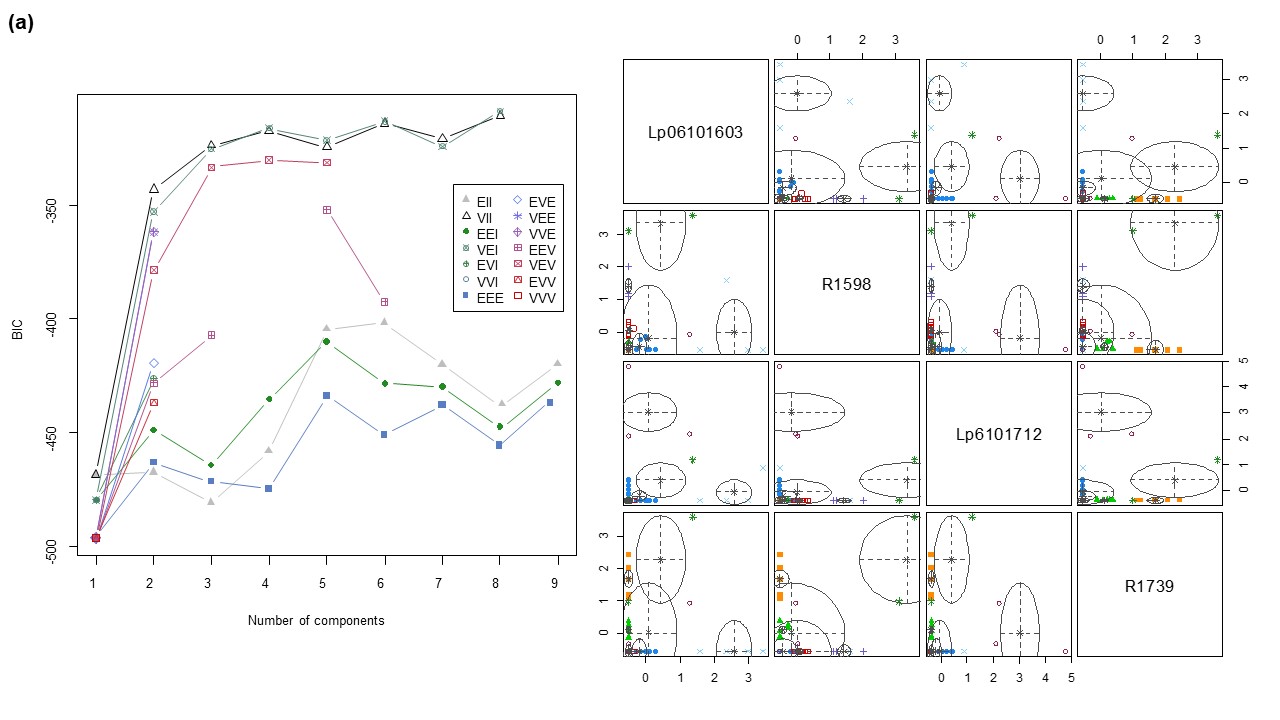

Supplement: Supplementary file 1 [file antibiotics-11-01844-s001.zip › FigureS6a.jpg]

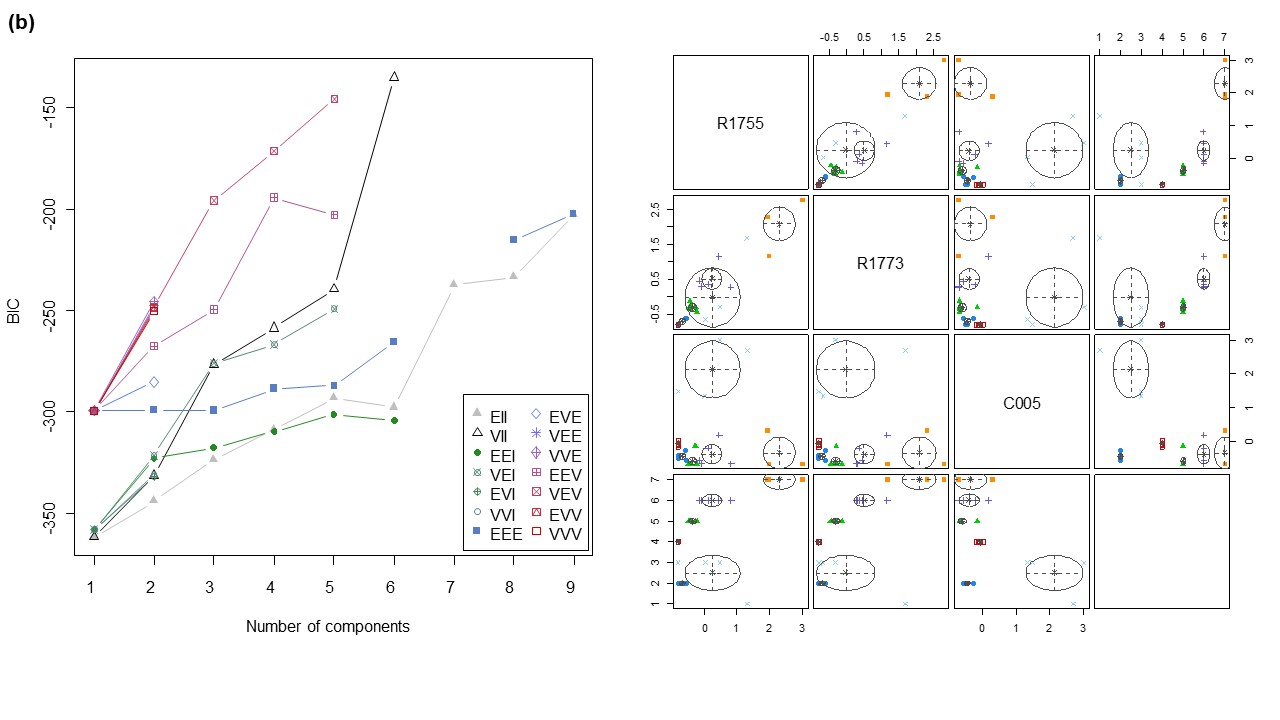

Supplement: Supplementary file 1 [file antibiotics-11-01844-s001.zip › FigureS6b.jpg]

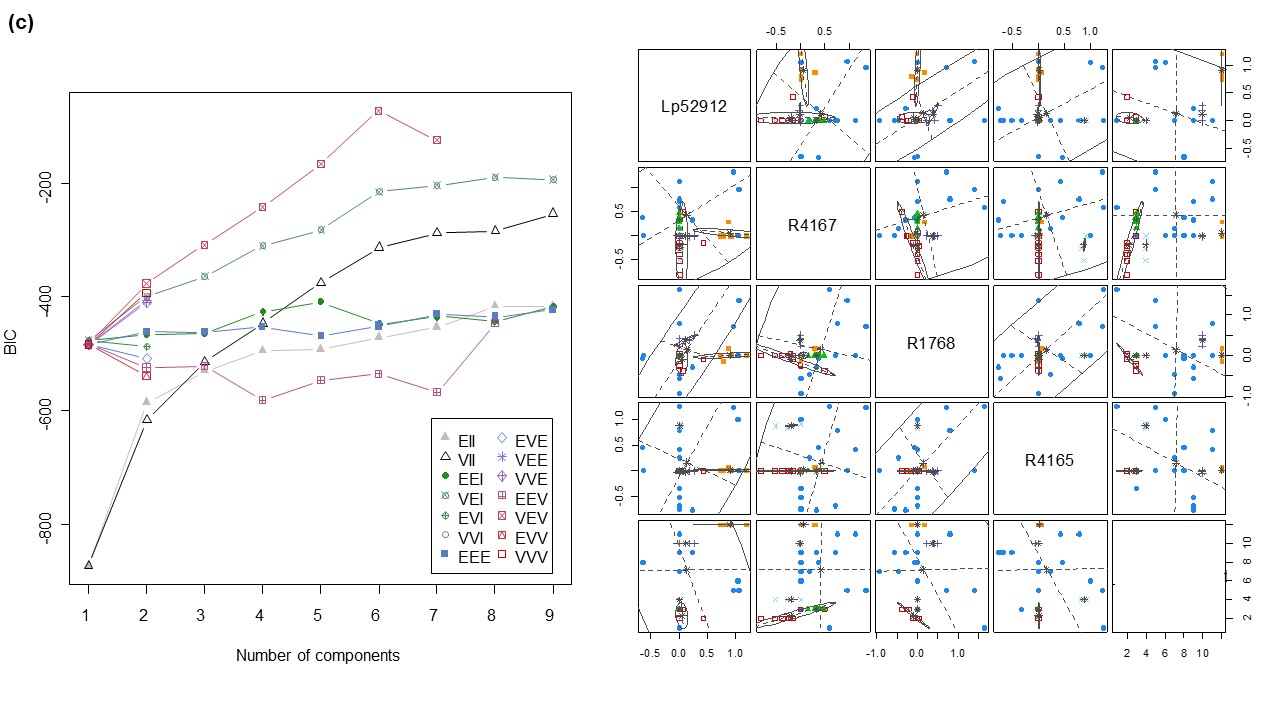

Supplement: Supplementary file 1 [file antibiotics-11-01844-s001.zip › FigureS6c.jpg]

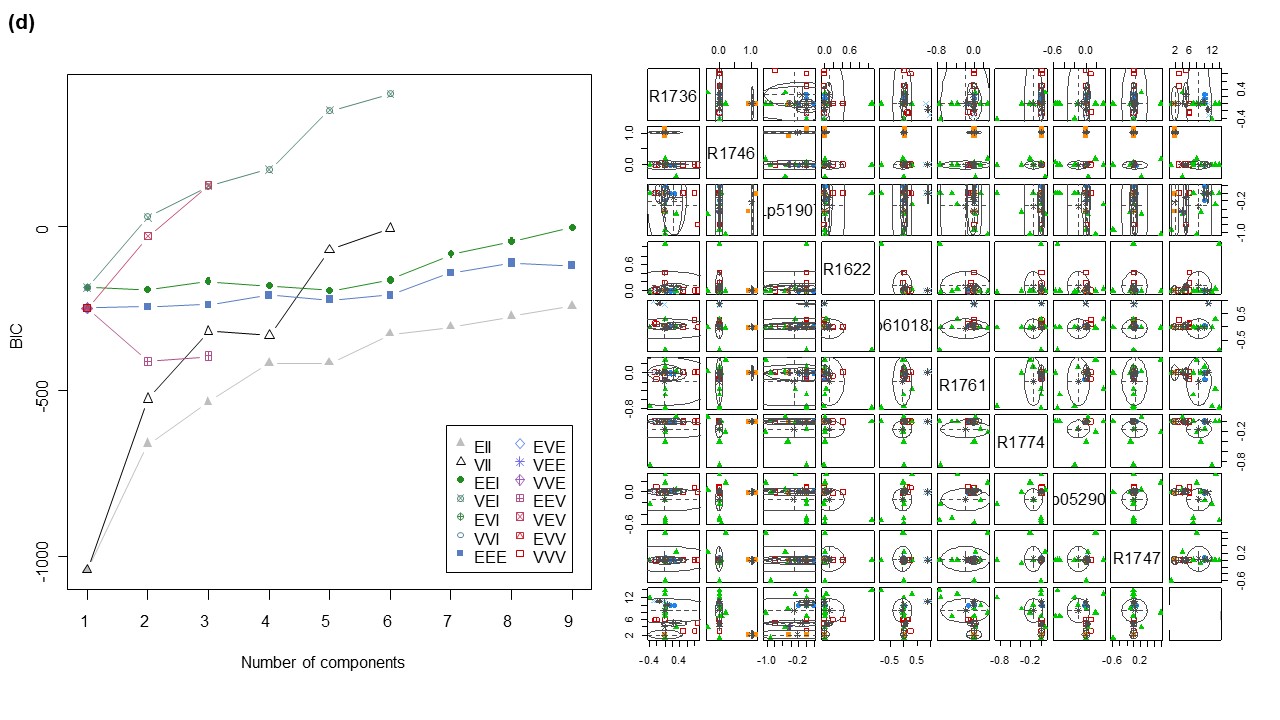

Supplement: Supplementary file 1 [file antibiotics-11-01844-s001.zip › FigureS6d.jpg]

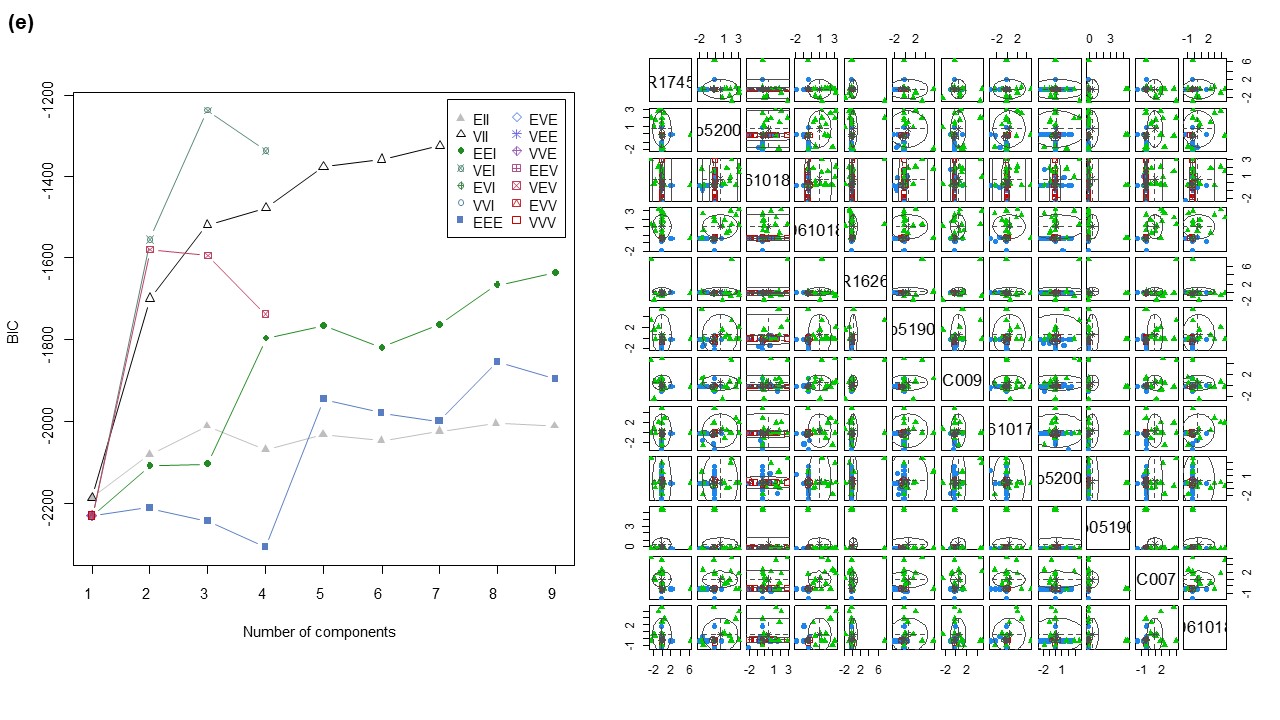

Supplement: Supplementary file 1 [file antibiotics-11-01844-s001.zip › FigureS6e.jpg]

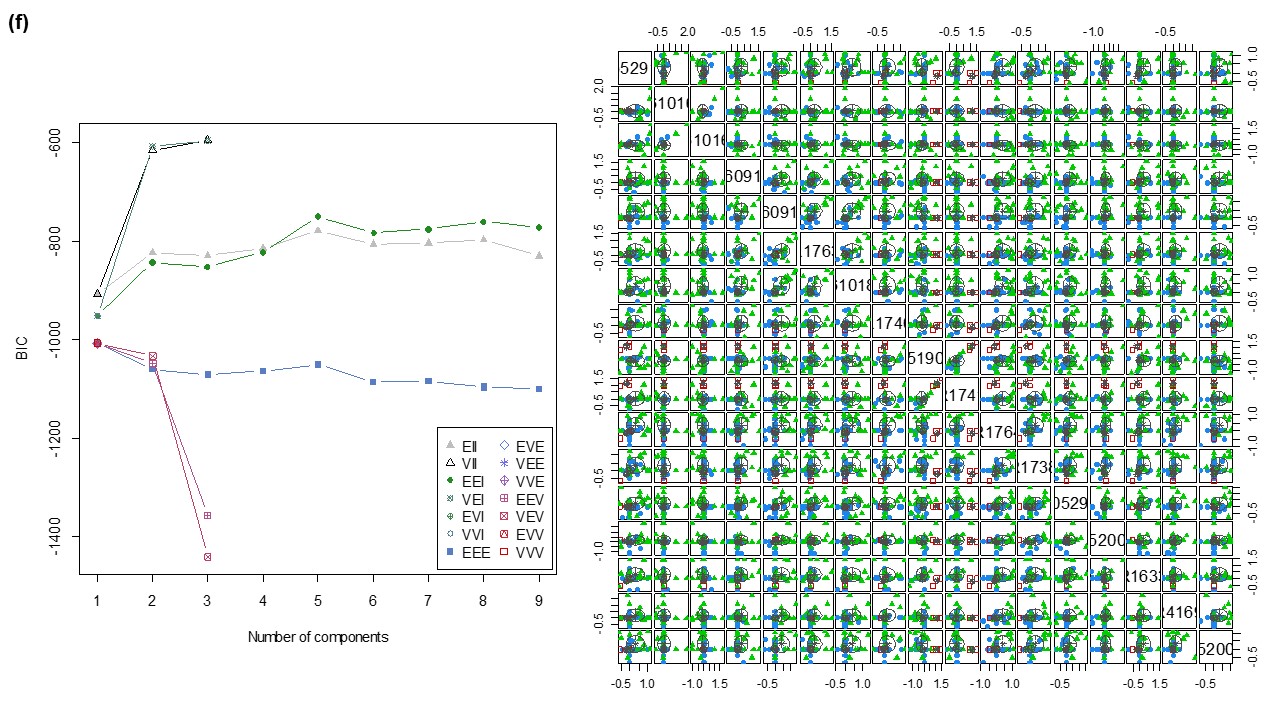

Supplement: Supplementary file 1 [file antibiotics-11-01844-s001.zip › FigureS6f.jpg]

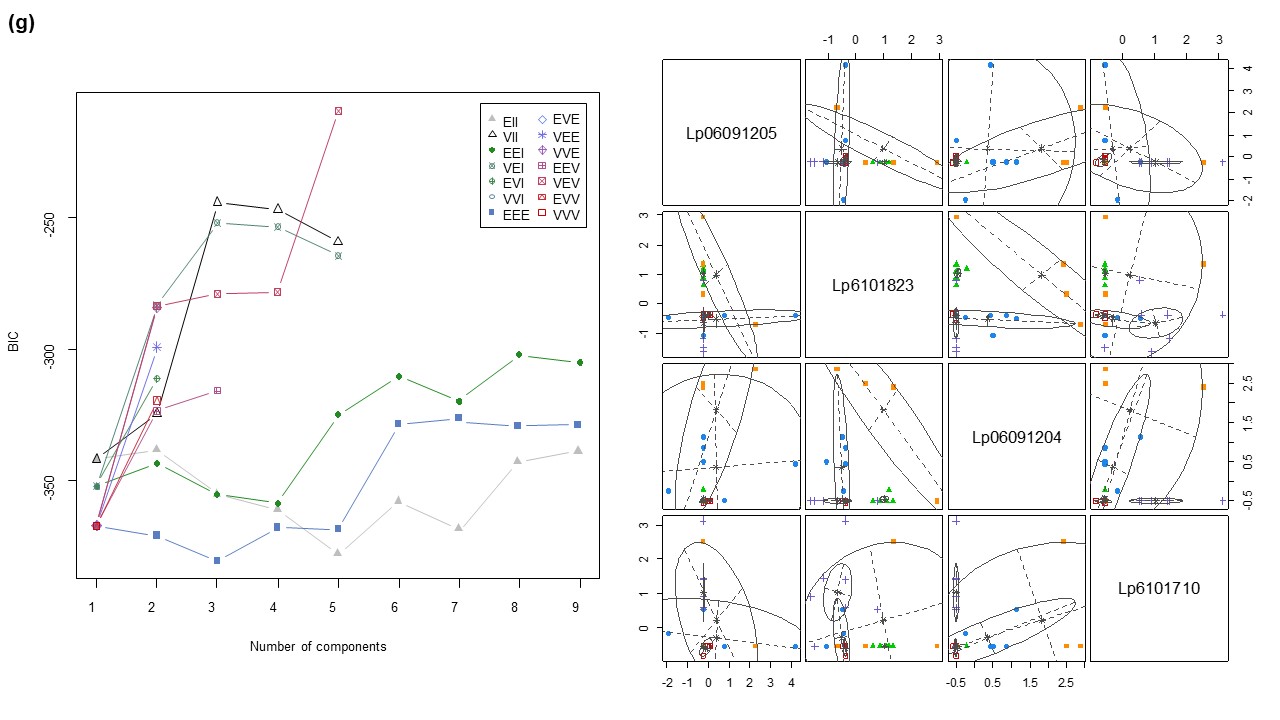

Supplement: Supplementary file 1 [file antibiotics-11-01844-s001.zip › FigureS6g.jpg]

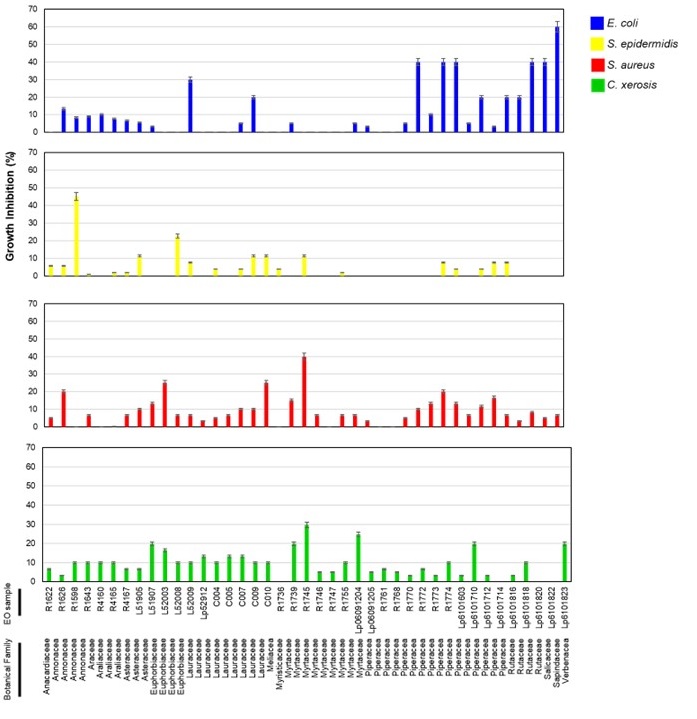

Supplement: Supplementary file 1 [file antibiotics-11-01844-s001.zip › FigureS7.jpg]
